# Ecosystem response persists after a prolonged marine heatwave

**DOI:** 10.1038/s41598-021-83818-5

**Published:** 2021-03-18

**Authors:** Robert M. Suryan, Mayumi L. Arimitsu, Heather A. Coletti, Russell R. Hopcroft, Mandy R. Lindeberg, Steven J. Barbeaux, Sonia D. Batten, William J. Burt, Mary A. Bishop, James L. Bodkin, Richard Brenner, Robert W. Campbell, Daniel A. Cushing, Seth L. Danielson, Martin W. Dorn, Brie Drummond, Daniel Esler, Thomas Gelatt, Dana H. Hanselman, Scott A. Hatch, Stormy Haught, Kris Holderied, Katrin Iken, David B. Irons, Arthur B. Kettle, David G. Kimmel, Brenda Konar, Kathy J. Kuletz, Benjamin J. Laurel, John M. Maniscalco, Craig Matkin, Caitlin A. E. McKinstry, Daniel H. Monson, John R. Moran, Dan Olsen, Wayne A. Palsson, W. Scott Pegau, John F. Piatt, Lauren A. Rogers, Nora A. Rojek, Anne Schaefer, Ingrid B. Spies, Janice M. Straley, Suzanne L. Strom, Kathryn L. Sweeney, Marysia Szymkowiak, Benjamin P. Weitzman, Ellen M. Yasumiishi, Stephani G. Zador

**Affiliations:** 1 Alaska Fisheries Science Center, National Oceanic and Atmospheric Administration, Juneau, AK USA; 2 U.S. Geological Survey, Alaska Science Center, Juneau, AK USA; 3 National Park Service, Fairbanks, AK USA; 4 University of Alaska Fairbanks, Fairbanks, AK USA; 5 Alaska Fisheries Science Center, National Oceanic and Atmospheric Administration, Seattle, WA USA; 6 Marine Biological Association, Nanaimo, BC Canada; 7 Prince William Sound Science Center, Cordova, AK USA; 8 U.S. Geological Survey, Alaska Science Center, Anchorage, AK USA; 9 Alaska Department of Fish and Game, Juneau, AK USA; 10 Pole Star Ecological Research LLC, Anchorage, AK USA; 11 U.S. Fish and Wildlife Service, Homer, AK USA; 12 Institute for Seabird Research and Conservation, Anchorage, AK USA; 13 Department of Fish and Game, Cordova, AK USA; 14 National Ocean Service, National Oceanic and Atmospheric Administration, Homer, AK USA; 15 U.S. Fish and Wildlife Service, Anchorage, AK USA; 16 Alaska Fisheries Science Center, National Oceanic and Atmospheric Administration, Newport, OR USA; 17 Alaska SeaLife Center, Seward, AK USA; 18 North Gulf Oceanic Society, Homer, AK USA; 19 University of Alaska Southeast, Sitka, AK USA; 20 Shannon Point Marine Center, Western Washington University, Anacortes, WA USA

**Keywords:** Ecology, Climate sciences, Ecology, Ocean sciences

## Abstract

Some of the longest and most comprehensive marine ecosystem monitoring programs were established in the Gulf of Alaska following the environmental disaster of the *Exxon Valdez* oil spill over 30 years ago. These monitoring programs have been successful in assessing recovery from oil spill impacts, and their continuation decades later has now provided an unparalleled assessment of ecosystem responses to another newly emerging global threat, marine heatwaves. The 2014–2016 northeast Pacific marine heatwave (PMH) in the Gulf of Alaska was the longest lasting heatwave globally over the past decade, with some cooling, but also continued warm conditions through 2019. Our analysis of 187 time series from primary production to commercial fisheries and nearshore intertidal to offshore oceanic domains demonstrate abrupt changes across trophic levels, with many responses persisting up to at least 5 years after the onset of the heatwave. Furthermore, our suite of metrics showed novel community-level groupings relative to at least a decade prior to the heatwave. Given anticipated increases in marine heatwaves under current climate projections, it remains uncertain when or if the Gulf of Alaska ecosystem will return to a pre-PMH state.

## Introduction

Understanding how marine ecosystems respond to cyclical, linear, or random environment change, or their additive effects, is a key challenge in marine ecology and resource management. Marine biological regime shifts^1^ of various magnitudes have been documented globally^2,3^. The Gulf of Alaska (GOA) has undergone one well-defined and sustained ecosystem regime shift^4,5^, and several others that were less evident and did not persist^6,7^. Regime shifts in large marine ecosystems such as the GOA are often correlated with basin-scale climate variables^3^ such as the Pacific Decadal Oscillation^8^, El Niño Southern Oscillation^9^, or North Pacific Gyre Oscillation^10^. The strength or direction of these climate-biology relationships, however, can vary through time^11–13^.

As physical climate indicators return to previous levels, the ecosystem might respond by returning to a previous state, but this is not always the case^5^. There are several examples from the northeast Pacific Ocean^7,14^ where key biological indicators began trending back toward a previous state, but ultimately were not sustained^15^. These occurrences are in part a result of time-varying climate-biology relationships^12,16^. Given recent trends in global climate patterns^17^, it is becoming more likely that current and future transitions may not shift back to prior regimes, but instead may produce novel ecosystem states.

A key ecosystem driver in contemporary climate states is marine heatwaves. Marine heatwaves are becoming more frequent and intense worldwide^18^. There has been an increase from 30% in 2012 to nearly 70% of global oceans in 2016 experiencing strong or severe heatwaves and the breadth of their impact is evidenced by a two-order-of-magnitude increase in scientific publications on marine heatwaves during the past decade^19^. There has also been an increase in documentation of disturbance to marine ecosystems, biodiversity, and ecosystem services associated with marine heatwaves^20^.

**Figure 1:**
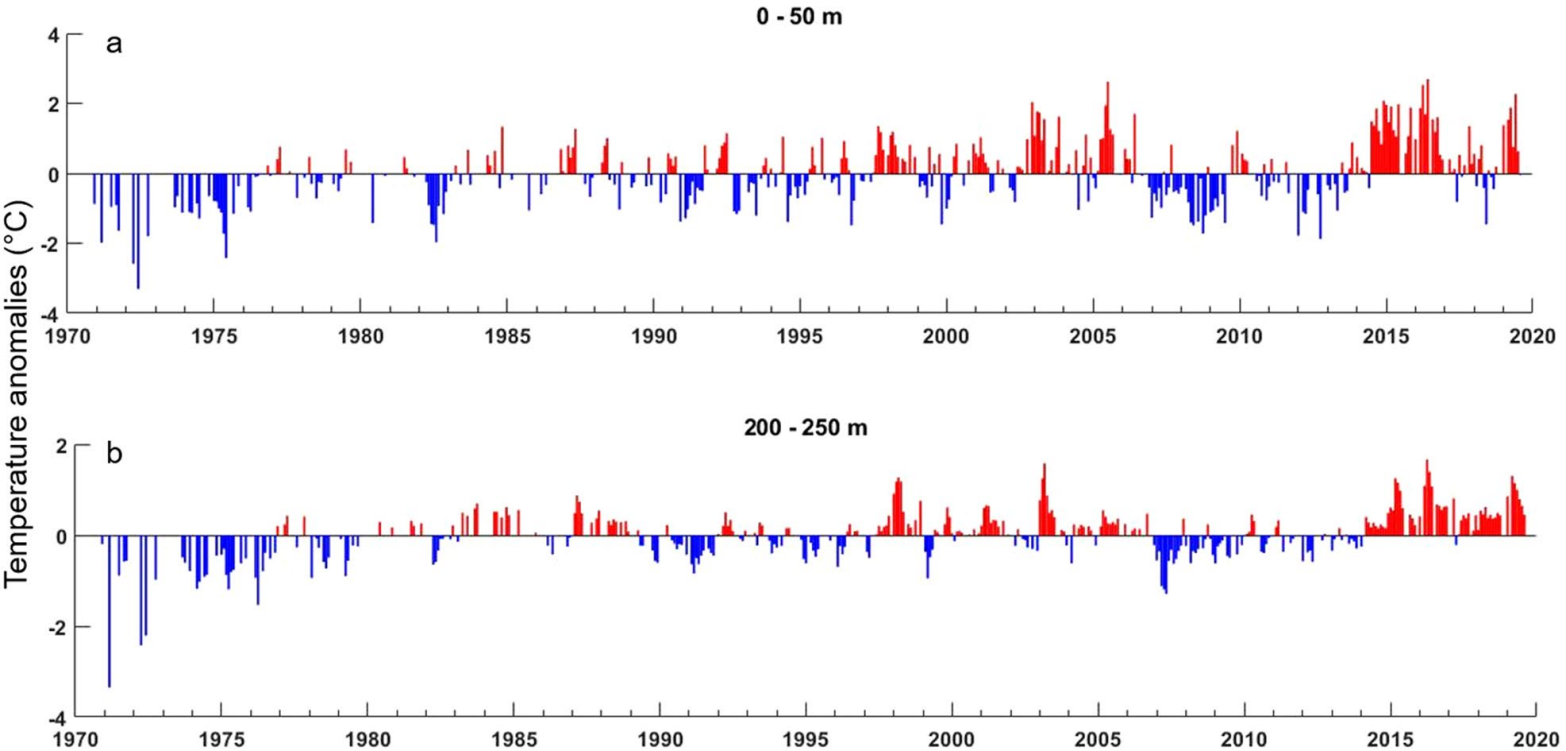
The multi-year heatwave that began in 2014 was the most persistent in the 48-year time series and it extended throughout the water column of the continental shelf. Temperature anomalies (°C) of the (**a**) upper (0–50 m) and (**b**) lower (200–250 m) water column at the GAK1 oceanographic station in the Gulf of Alaska, 1973–2019 (for location, see Fig. 2).

The northeast Pacific marine heatwave (PMH) that peaked in 2015 was particularly notable in its magnitude and spatio-temporal extent. The PMH lasted two years^21^ and was the only marine heatwave lasting through all four seasons, which was 2–10 times longer than any other heatwave recorded globally in the past decade^19^. In mid-2016, the PMH began to dissipate, based on satellite-derived sea surface temperature data^19^. While in situ sea surface temperatures in some areas of the GOA did trend back toward pre-PMH conditions, water column and bottom temperature anomalies remained strongly positive down to at least 250 m (Fig. 1)^22^. The hiatus in the surface expression of the PMH, however, was short-lived and the warming re-intensified in late-2018 and persisted into fall 2019 (Fig. 1)^23^.

Biological responses to the 2014–2016 PMH were diverse and occurred throughout the North Pacific, including species range shifts^24^, changes in lower trophic level community composition^25,26^, predator mortality events^27,28^, and effects on commercial fisheries, including aquaculture^29^. However, initial and potential multi-year lingering effects of the PMH have not previously been quantitatively evaluated in a fully integrated, ecosystem approach.

**Figure 2:**
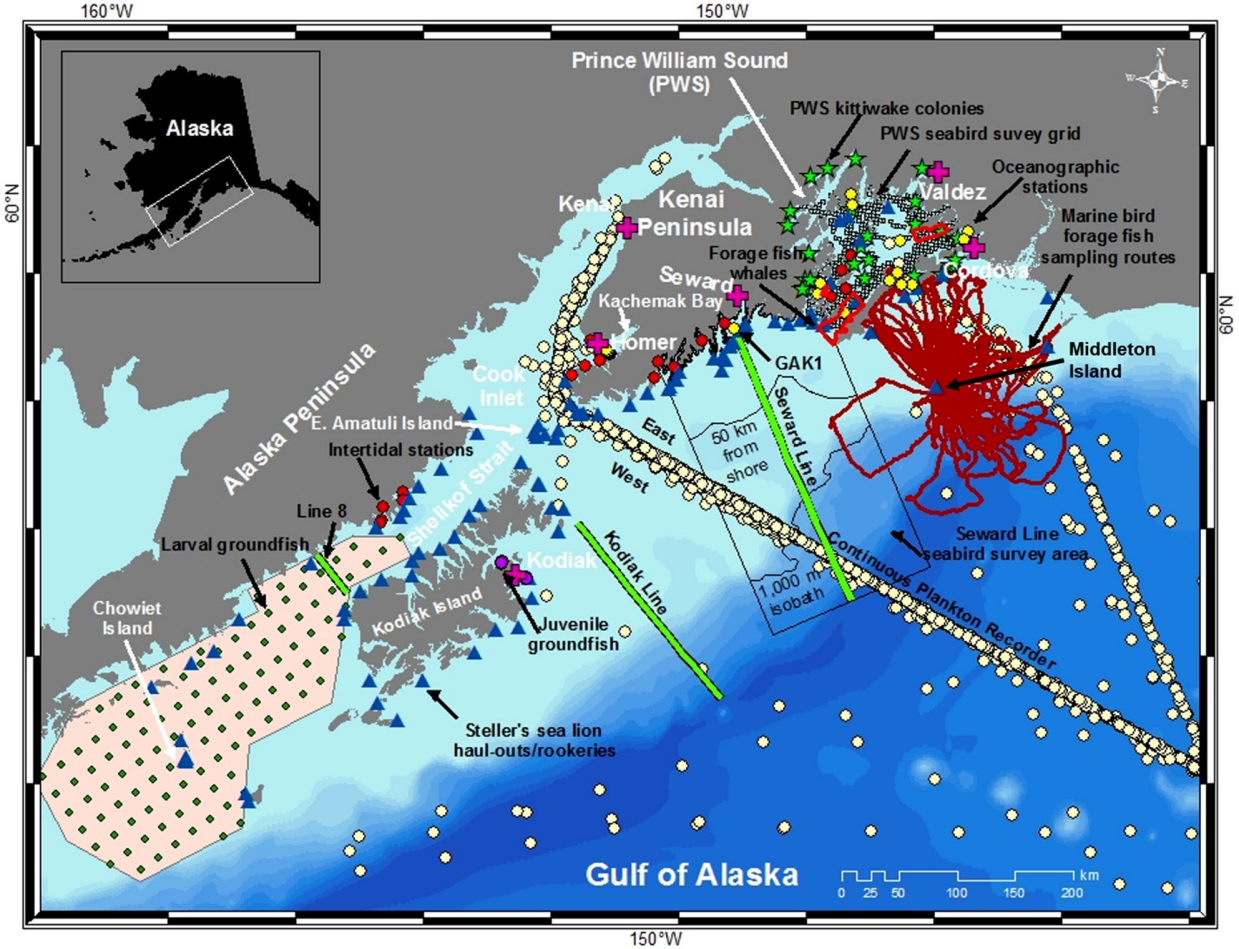
Sampling locations in the northern Gulf of Alaska. The division for references to eastern and western study area is the continuous plankton recorder transect into Cook Inlet. The black contours within the Seward Line marine bird survey area differentiate inner continental shelf (shore to 50 km from shore), middle shelf (50 km from shore to shelf-slope break, defined using 1000 m isobath) and oceanic (seaward of the 1000 m isobath) domains. The 1000 m isobath is also used to distinguish shelf versus oceanic zooplankton samples from the continuous plankton recorder. Marine bird foraging routes provided by Dr. Shannon Whelan, McGill University. This map was created using ArcGIS software (ArcMap 10.7.1; www.esri.com).

The Gulf of Alaska has extensive long-term monitoring programs, many of which were established in response to the *Exxon Valdez* oil spill and the need to assess long-term recovery of injured resources given highly variable environmental conditions^30,31^. We leveraged data from this wealth of long-term monitoring programs to assess how the northern GOA ecosystem, including intertidal to oceanic domains and primary production to commercial fisheries (Fig. 2), responded to the PMH and subsequent cooling and warming events. We used 187 time series of annual biological metrics (Table 1) to determine: (1) how the biological community responded as a whole; (2) which taxa or metrics exhibited negative, positive, or neutral responses; and (3) whether taxa or metrics showed continued, multi-year response or signs of recovery for up to 5 years after the onset of the PMH.

**Table 1:** Type, number, and months sampled of biological time series (n = 187 total) used to assess response to a marine heatwave in the Gulf of Alaska. Sources of time series include the *Exxon Valdez* Oil Spill Trustee Council's (EVOSTC) Gulf Watch Alaska (n = 77), NOAA Alaska Fisheries Science Center (n = 58), U.S. Fish and Wildlife Service (n = 22), Northern Gulf of Alaska long-term ecological research site (n = 18), Alaska Department of Fish and Game (n = 8), Alaska SeaLife Center (n = 2), and the *EV* OSTC Herring Research and Monitoring Program (n = 2). See Table S1for more details on time series metrics.

Ecosystem component	# of time series	Months sampled	Type, taxa, or life stage
Phytoplankton	10	Apr–Oct	Satellite-derived biomass (n = 4) and phenology (n = 2), in situ size (n = 4)
Zooplankton	27	Jan–Dec	Microzooplankton biomass (n = 4), microzooplankton fraction of ciliates (n = 4), warm water/cool water/individual species biomass (n = 13), copepod community size (n = 6)
Intertidal	14	May–July	Mussel (n = 4), sea star (n = 4), *fucus* (n = 4)
Forage fish	14	May–Aug	Herring/sand lance/capelin/hexagrammid abundance (n = 12), condition (n = 2)
Groundfish	12	May–Sep	Pollock/sole/cod/flounder/sablefish early life stages (n = 8), adult (n = 4)
Marine birds	52	Feb–Dec	Seabird/shorebird/sea duck abundance (n = 39), productivity (n = 13)
Marine mammals	16	May–Sep	Cetacean (n = 3) and pinniped (n = 10) abundance, sea otter foraging success (n = 3)
Commercial Harvest	46	Jan–Dec	Salmon (n = 22) and groundfish (n = 24) quantity landed and revenue

## Results

### Common trends in biological observations

**Figure 3:**
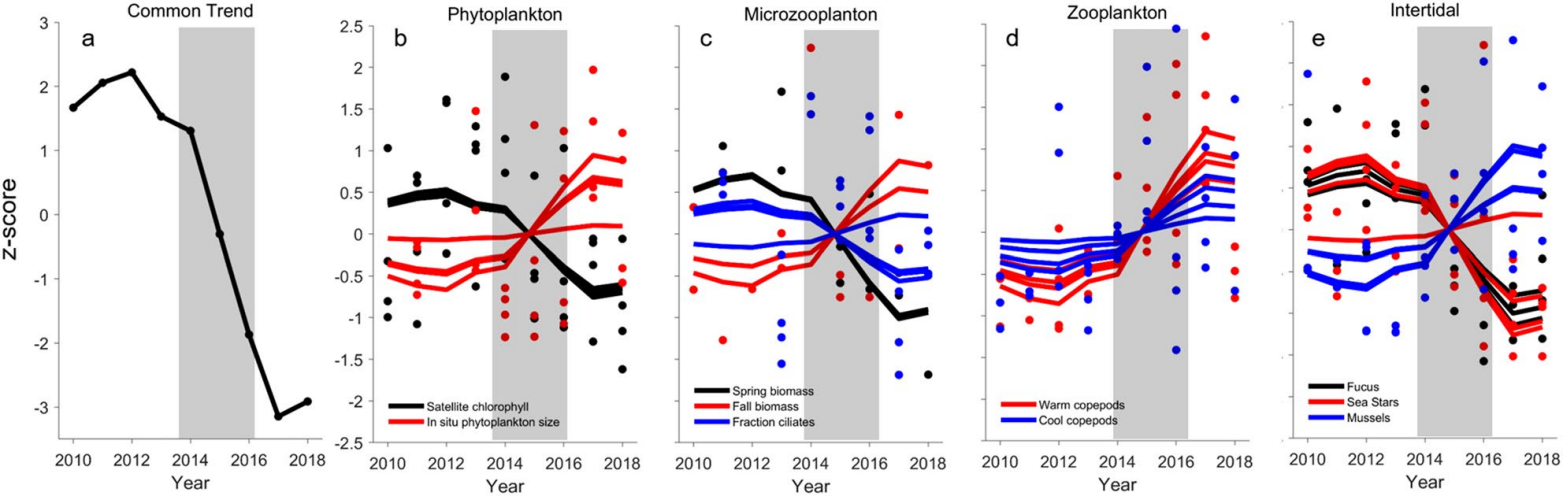
Response of lower trophic level species during the Pacific marine heatwave. (**a**) The common trend from the best model fit (lowest AICc; Table S2) identified from dynamic factor (DFA) analysis of 187 biological time series. (**b**) Phytoplankton indexed by satellite-derived chlorophyll biomass along the Seward and Kodiak oceanographic sampling lines and in situ measures of phytoplankton size composition from Seward Line sampling. (**c**) Microzooplankton seasonal biomass and fraction of ciliates from Seward Line sampling. (**d**) Zooplankton abundance for warm and cool water associated copepod species from continuous plankton recorder over oceanic and shelf waters and inside waters of Kachemak Bay and Prince William Sound. (**e**) Intertidal algal, sea star, and mussel abundance from four regions across the northern GOA. Points are annual values and solid line is DFA model fit to each time series. Grey shading represents the 2014–2016 northeast Pacific marine heatwave. Values are z-score standardizations so the y-axes are unitless.

Of the nine dynamic factor analysis models evaluated, the model with the lowest Akaike’s Information Criterion corrected for small sample sizes (AICc) included equal variance and covariance structure and one common trend (Table S2). The common trend line shows a steep decline starting in 2014 coincident with the PMH and continuing through 2017 with a slight increase in 2018, but still remaining well below pre-PMH levels (Fig. 3a).

Half of the time series (n = 98, 52%) had factor loadings indicating a strong negative or positive relationship to the trend line (≥ 0.20, absolute value), whereas the other half of the time series (n = 89, 48%) had marginal or weak factor loadings (< 0.20, absolute value) and were identified as a “neutral” response (Tables 2, S1). The direction of response among time series, however, varied by taxon. Time series with the highest percentage of negative responses were phytoplankton (40%), intertidal organisms (50%), and commercial harvest (50%; Table 2). Zooplankton had primarily positive (warm water associated species) or neutral (microzooplankton and cool water associated species) responses and forage fish responses were evenly distributed among negative (33%), positive (33%), and neutral (33%). Groundfish, marine bird, and marine mammal metrics primarily exhibited neutral responses (50%-65%; Table 2). Notable exceptions within these categories include primarily negative responses of adult groundfish populations, colony-based metrics for piscivorous seabirds in our eastern study area, abundance of humpback whales, and haul-out or rookery-based metrics for pinnipeds in our eastern study area (Tables 2, S1).

**Table 2:** Summary of trends based on factor loadings from dynamic factor analysis (DFA) by taxa and metric of 187 biological time series.

Taxa and metric	Negative n (%)	Positive n (%)	Neutral n (%)
Phytoplankton	4 (40%)	3 (30%)	3 (30%)
Abundance	3		1
Size		3^a^	1
Phenology	1		1
Zooplankton	4 (15%)	12 (44%)	11 (41%)
Micro	2	1	5
Cool		2	3
Warm		5	
All		2	
Size	2	2	2
Jellies			1
Intertidal	7 (50%)	3 (21%)	4 (29%)
Algae	4		
Invertebrates	3	3	4
Forage fish	4 (33%)	4 (33%)	4(33%)
Abundance	3	4	3
Performance/condition	1		1
Groundfish	3 (25%)	3 (25%)	6 (50%)
Early life stage		3^a^	5
Adult	3		1
Marine birds	11 (21%)	7 (14%)	34 (65%)
Colony	5	1	12
At-sea	6	6	22
Marine mammals	5 (31%)	2 (13%)	9 (56%)
Otters	1		2
Pinnipeds	3	2	5
Whales	1		2
Commercial harvest	22 (50%)	5 (11%)	17 (39%)
Chinook	1		1
Coho		1	1
Sockeye	8		4
Pink			2
Chum			2
Groundfish	13	4	7
Grand total	59	39	89

Each time series is described as having a negative, positive, or neutral response (trend) coincident with the 2014–2016 Pacific Marine Heatwave. Negative, positive, or neutral responses are determined by DFA loadings of ≥ 0.20, ≤ -0.20, or > -0.20 and < 0.20, respectively. The signs of factor loadings are opposite of time series response because factor loadings describe how a given time series relates to the common trend, which was negative during the PMH (Fig. 3a). See Table S1 for factor loadings of individual time series

^a^cell size of phytoplankton and abundance of juvenile pollock declined during strongest heatwave years, but increased above pre-heatwave levels resulting in a positive model trend overall.

### Lower trophic levels

Phytoplankton showed a general decrease in surface biomass (chlorophyll) during the PMH. The decrease in cell size during the PMH was followed by an abrupt increase following the PMH, resulting in a positive model fit (Fig. 3b). Microzooplankton trends varied by season and metric with a decrease in spring biomass and an increase the fraction of ciliates during the PMH, followed by a rapid decline, and an increase in fall biomass (Fig. 3c). In contrast, zooplankton abundance trends were primarily positive or neutral during the PMH. Warm-water associated zooplankton species increased markedly during the PMH, with the increase most distinct in continental shelf waters, Kachemak Bay, Prince William Sound, and less so in oceanic waters (Fig. 3d, Table S1). There was some variation in the timing of increase among domains; however, most anomalies stayed positive even as the upper water column cooled in 2017 and finally returned to pre-PMH levels in 2018. Surprisingly, the abundance of cool-water associated zooplankton did not show a consistent declining trend regardless of domain, but instead generally showed no trend or a temporary increase.

Intertidal taxa showed remarkably consistent trends among spatially replicated sampling areas throughout the northern GOA. Algal (*Fucus*) cover declined precipitously with some variability in timing in all areas (Fig. 3e). Similarly, the abundance of sea stars—key intertidal predators—declined precipitously after the onset of the PMH in most areas. In contrast, the abundance of mussels, a main sea star prey item, increased in most areas (Fig. 3e) and in the diets of glaucous-winged gulls (Table S1).

### Middle and upper trophic levels

**Figure 4:**
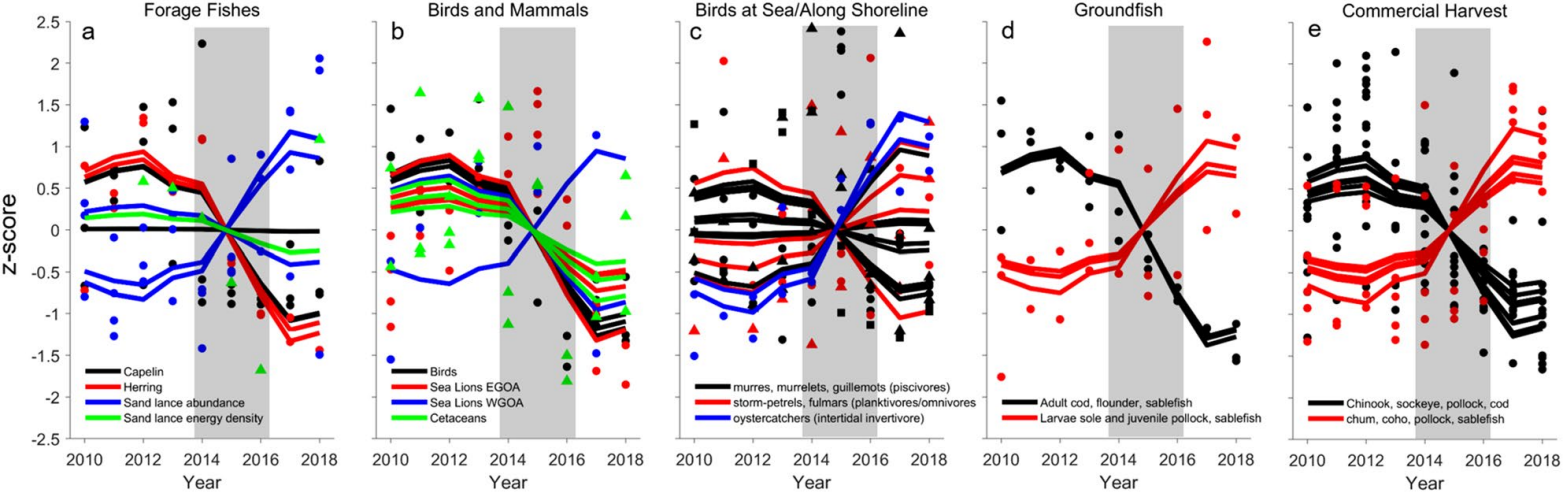
Response of mid and upper trophic level species during the Pacific marine heatwave. (**a**) Abundance of juvenile capelin and sand lance from marine bird diets and of herring spawn, herring age-3 growth, and sand lance body condition in Prince William Sound (PWS). (**b**) Abundance of nesting murres on East Amatuli Island, kittiwake nests in Prince William Sound (“birds”), Steller sea lion pups and non-pups on rookeries and haul-out sites in eastern and western Gulf of Alaska (GOA), humpback and killer whale encounter rates, and killer whale numbers (“cetaceans”). (**c**) Marine bird abundance of primarily piscivores including common murres (black dots) from PWS and the Seward Line, murrelets (black triangles) from PWS, Kenai and Alaska Peninsulas, and pigeon guillemots (black squares) from Kenai and Alaska Peninsulas; primarily planktivores/omnivores including storm-petrels (red dots) and northern fulmars (red triangles) along the Seward Line; and intertidal invertebrate consumers—black oystercatchers—from Kenai and Alaska Peninsulas. (**d**) Adult female spawning biomass of Pacific cod, arrowtooth flounder, and sablefish, and larval southern rock sole, juvenile pollock, and growth of juvenile sablefish. (e) Commercial harvest in pounds landed and ex-vessel revenue for salmon and groundfish species from regions throughout our eastern and western study areas. Points are annual values and lines are models fit to data. Grey shading represents the 2014–2016 northeast Pacific marine heatwave. Values are z-score standardizations so the y-axes are unitless.

Trends in two forage fish species, capelin abundance in the eastern study area and herring abundance and growth in Prince William Sound (PWS), decreased markedly and remained at consistently low values after the onset of the PMH (Fig. 4a). The decline in capelin was evident in diets of both surface-feeding and diving birds. The abundance of sand lance larvae in the western and juveniles in eastern study area showed an opposite trend of marked increase during the PMH. In contrast, capelin and sand lance abundance in seabird diets from the western study area and age-1 sand lance whole body energy showed neutral to slight declining trends (Fig. 4a).

Marine bird colony metrics and mammal abundance trends, especially for piscivorous species in the eastern study area, were generally negative during the PMH (Fig. 4b). The abundance of nesting common murres at East Amatuli Island, kittiwake nests in PWS, breeding success in PWS and Middleton Island, and Steller sea lion pup counts on rookeries all declined. Parallel declines also occurred for combined counts of adult, sub-adult, and juvenile sea lions (“non-pups”) in the eastern portion of our study area, but not the western (Fig. 4b). Chiefly planktivorous, omnivorous, or generalist species such as fork-tailed storm-petrels, parakeet auklets, glaucous-winged gulls, pelagic cormorants, and rhinocerous auklets—especially in the western study area—showed primarily neutral responses. Humpback and killer whale abundance (indexed by encounter rate or group size) in the PWS region also declined after the onset of the PMH, but the decline was only sustained for humpback whales through 2018 (Fig. 4b; Table S1).

Marine bird abundance at sea showed a mix of response trends and short-term, annual variation. The abundance responses of primarily piscivorous species in summer and fall were neutral (murres) or declined (murrelets and guillemots), with one exception of an increase in murrelets during winter in PWS (Fig. 4c; Table S1). Murres showed more short-term variation, especially with an influx into nearshore areas of PWS and Kenai and Alaska Peninsulas during the PMH in 2015 (Fig. 4c individual points). Primarily zooplanktivorous or omnivorous storm-petrels and fulmars increased in middle- and inner-shelf areas and declined in offshore areas during the PMH, with the response strongest and most persistent for storm-petrels (Fig. 4c; Table S1). The abundance of black oystercatchers that feed on intertidal invertebrates increased sharply in the eastern and western GOA areas surveyed.

Response trends in groundfish varied by adult versus younger age classes. Significant negative trends were primarily found in adult female spawning biomass of Pacific cod, sablefish, and arrowtooth flounder (Fig. 4d); and neutral response by pollock (Table S1). Positive trends occurred for abundance of larval southern rock sole and juvenile pollock, and growth rate of juvenile sablefish (Fig. 4d). Although juvenile pollock declined during the PMH, increases post-PMH led the model to fit an increasing trend through 2018. Changes in abundance of pollock and northern rock sole larvae, and of Pacific cod larvae and juveniles were initially negative and saffron cod positive, but these trends were not sustained; this resulted in overall neutral trends (Table S1).

Commercial harvest trends in pounds and revenue were strongly negative for Pacific cod delivered to fishing ports throughout our study area, sockeye salmon for ports in the eastern study area and Chinook salmon in the west (Fig. 4e). Pollock harvest showed a negative trend at one port in the east and positive at one port in the west. There were fewer positive trends in harvest, primarily chum salmon and sablefish at a couple of ports in the east, and coho salmon and pollock at Kodiak in the west (Fig. 4e; Table S1).

### Community analysis

**Figure 5:**
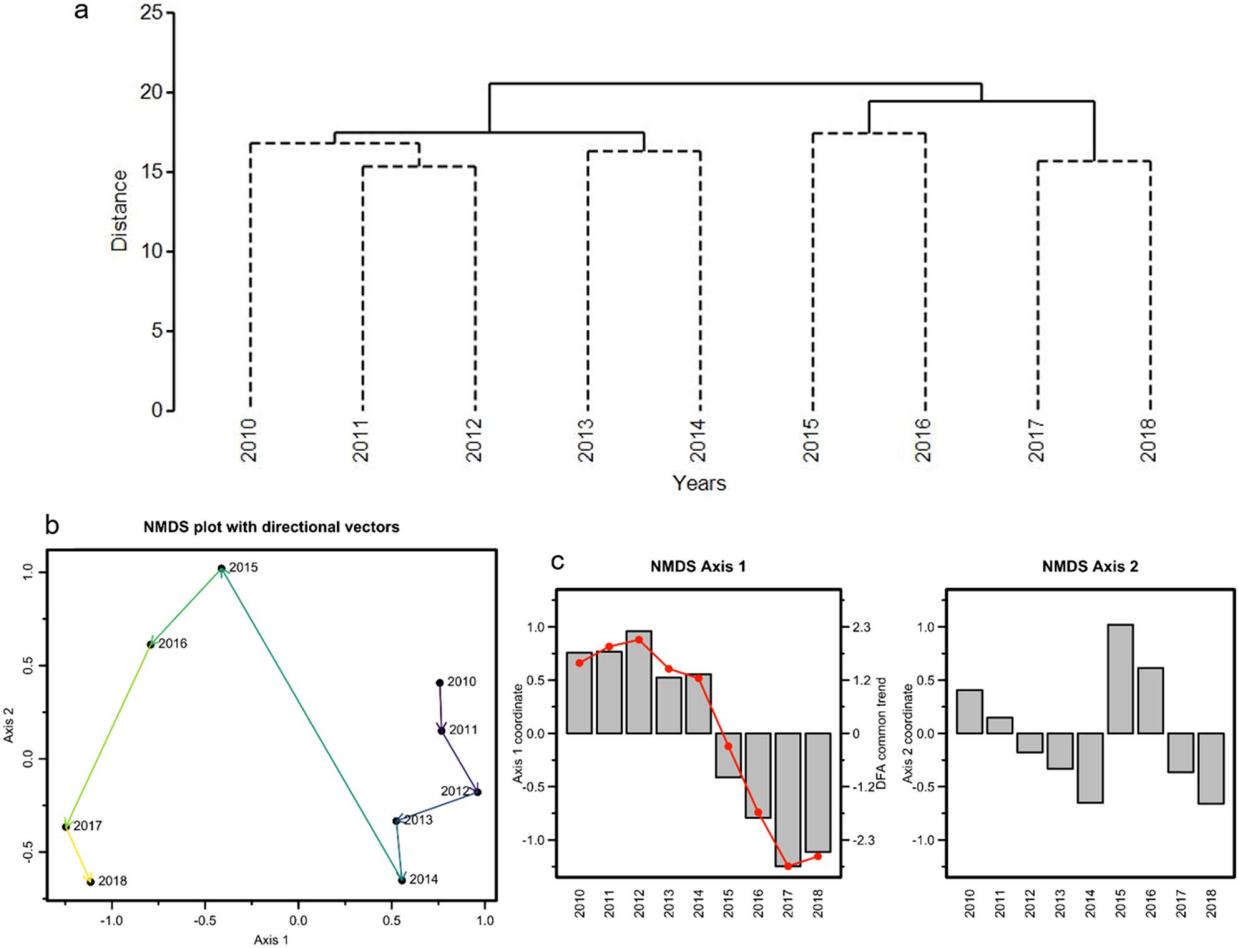
Community analyses of 187 biological time series in the Gulf of Alaska. (**a**) Cluster analysis showing significantly different groupings (solid lines). (**b**) Non-metric multi-dimensional scaling (nMDS) analysis of annual changes in Gulf of Alaska community prior to and during a multi-year marine heatwave (strongest from 2014–2016) in the Gulf of Alaska (2D stress 0.08). (**c**) Temporal patterns in nMDS axes, with dynamic factor analysis common trend overlay (red line) on axis 1.

Annual patterns in the combined community composition of our 187 time series of the GOA ecosystem showed distinct groupings of before and after the onset of the PMH in both the cluster analysis (Fig. 5a) and non-metric multidimensional scaling ordination (nMDS, 2 dimension, stress = 0.08; Fig. 5b). Whereas years were more tightly clustered during a period of relatively cold ocean temperatures (2010–2013; Fig. 1) before the PMH, there was greater distance among years after the onset of the PMH (2015–2018; Fig. 5a, b). The largest changes in directional vectors of the nMDS occurred during 2014–2015 and 2016–2017 (Fig. 5b). The temporal pattern in axis 1 of the nMDS reflects the common trend identified by DFA (Fig. 5c), whereas axis 2 reflects species that responded strongly to the 2015–2016 maximal intensity of the heatwave, then reverted to pre-heatwave state. The DFA common trend was highly correlated with nMDS axis 1 (r = 0.99), indicating that both methods identified the same pattern of response to the PMH. Correlation of individual time-series with nMDS axis 1 therefore closely followed those previously discussed for DFA results (Fig. S1a). Time series that most strongly correlated (> 0.40) with initial response then return to pre-PMH levels described by nMDS axis 2 included phytoplankton phenology, phytoplankton size, and forage fish growth and condition (Fig. S1b).

Community analysis by region showed strong before-after PMH pattern overall, with slightly different year groupings in east versus west (Fig. S2a,b). Cluster analysis further differentiated the 9-year period before, during, and after the PMH into two (east) or three (west) significantly different groups (similarity profiles, p < 0.05), clearly differentiating 2010–2013 pre-PMH from 2014–2018 periods (Fig. S2,d).

Additional analyses confirmed that these results were not affected by missing values (0–20% of time series had missing values in any one year from 2010–2018) or by using a reduced length of time series. Rerunning the analyses with 93 time series with no missing values during 2013–2018 (some time series initiated before 2012, were not sampled in 2012) resulted in only one fewer year grouping in the cluster analysis, but no difference in results with respect to before and after the PMH.

### Longer time series perspective

**Figure 6:**
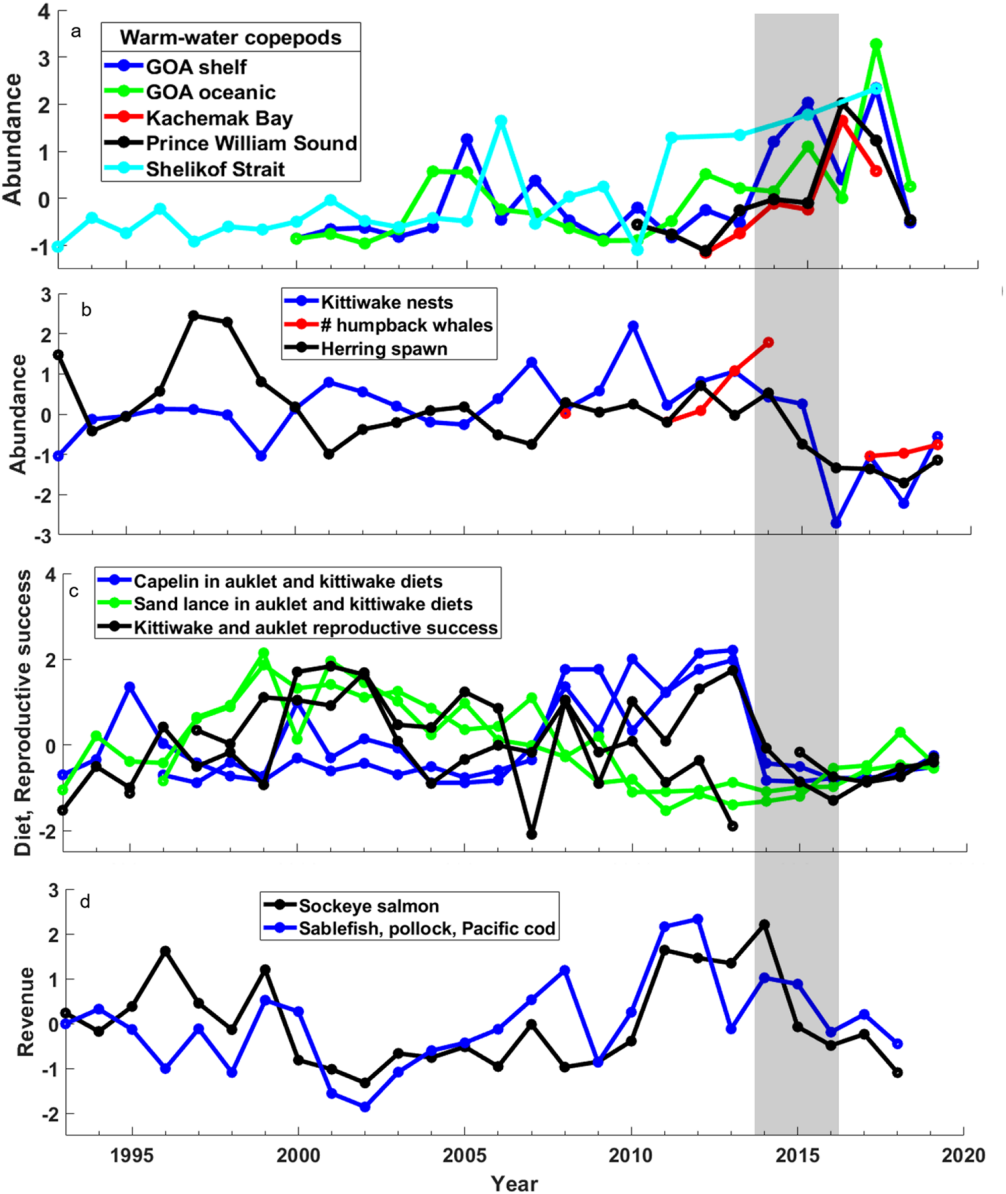
Long-term trends (1993–2019; **a**, **d** through 2018) in (**a**) the abundance of warm water associated copepod species in the Gulf of Alaska, (**b**) herring and herring-dependent predators in Prince William Sound, (**c**) capelin and sand lance availability to marine birds and reproductive success of kittiwakes foraging from Middleton Island, and (**d**) ex-vessel revenue for sockeye salmon and combined for the three most valuable groundfish species. Grey shading represents the 2014–2016 northeast Pacific marine heatwave. Values are z-score standardizations so the y-axes are unitless.

Our time series that extend for decades prior to the PMH demonstrate that the biological response in the northern GOA to this recent event was indeed anomalous. For example, we repeated our cluster analysis on a reduced number (n = 87) of our biological time series that spanned a 19-year period before, during, and after the 2014–2016 PMH. These longer time series confirmed that 2016–2018, in particular, is distinct from the previous 16 years (Fig. S3). Other examples include the abundance of warm-water associated zooplankton; while these increased during previous warm periods in the GOA (e.g., 2003–2006) and at least one location beginning to increase before the PMH, the magnitude and duration of increase during the PMH was unprecedented in the time series (Fig. 6a). Likewise, for over two decades, the abundance of Pacific herring, humpback whales, and nesting black-legged kittiwakes varied between periods of warm and cold, but all exhibited a precipitous decline and remained low through 2019, 5 years after the onset of the PMH (Fig. 6b). This was similarly true with the availability of sand lance and capelin, as well as with effects on reproductive success to both surface feeding and diving birds foraging from Middleton Island. The decline was notably consistent among all metrics, persisted for 5 years after the onset of the PMH (Fig. 6c), and was unlike previous years of warm and cool periods. Sockeye salmon and groundfish revenue declined during the PMH from high points prior to the heatwave, however, the low values were within the range of previous lows in the past 19 years and the decline in some groundfish species began before the PMH (Fig. 6d).

## Discussion

The PMH was a major ecosystem perturbation corresponding to widespread effects throughout the food web, from phytoplankton to fishing communities and from intertidal to oceanic domains. The dominant pattern identified from our 187-member biological time series was an abrupt change during the PMH; over half of our time series showed either significant positive or negative responses. Such immediate responses across trophic levels through to commercial harvests are particularly notable without accounting for potential multi-year response lags among age classes and life histories of such diverse organisms. This further highlights the magnitude of this ecosystem perturbation. Collectively, this led to novel biological community patterns within 2 years of the onset of the PMH that was distinct from that prevailing at least 4–14 years prior. Whereas some metrics began trending back toward pre-PMH, these new community patterns have remained for 4 years post-onset of the PMH. The PMH was particularly strong in not only the spatial extent, duration, and magnitude of warming^19^, but also in the depth of warming and the diversity of habitats affected—ranging from offshore oceanic to intertidal waters in glacial fjords^32^. Furthermore, while water temperatures in some areas trended back toward pre-heatwave levels in years following the 2014–2016 PMH, others did not or did so only intermittently. Warm water anomalies persisted for at least 4–5 years after the beginning of the PMH, especially in the lower water column of the continental shelf (Fig. 1)^32^. Therefore, various life stages of many organisms were still experiencing anomalously warm conditions in up to five subsequent years; this likely explains the lag in returning to pre-heatwave values for the GOA community as a whole.

### Biological community response during the PMH

Some patterns of response during the PMH emerged across taxa and food webs of various biological communities. Shorter-term responses occurred across all trophic levels in the form of changing phenology and cohort demographics, e.g., size, growth, and condition. Longer-term, persistent responses also were observed across all trophic levels, from phytoplankton to whales. For example, trends in several primary and secondary production metrics did not begin returning to pre-PMH levels until the second year of the 2017–2018 heatwave hiatus. Other taxa, including intertidal macroalgae, forage fish, and whale metrics showed little or no return to pre-heatwave levels, indicating carryover effects of the prolonged heatwave.

Three main forage fishes of pelagic communities in the GOA (capelin, herring, sand lance) showed declines in abundance or condition during the PMH, and two species—capelin and herring—remained at low abundance levels through 2019 in the eastern GOA (Fig. 6)^33^. The decline in size and total energy content of sand lance^34^ was particularly striking given that the occurrence of this species increased beginning in 2016 (Fig. 4) and during other prior warm events in the GOA^35^. While conditions in 2016 were presumably favorable for age-0 sand lance, the lower energy content and size/age truncation observed in sand lance in that year suggest this key forage fish also experienced overall negative impacts during the PMH. Indeed, by 2019, sand lance abundance still remained well below historically high values (Fig. 6). Large-scale reductions in these and other forage species in the northern GOA appeared to restrict energy transfer to upper trophic level species^33^, leading to large-scale mortality events^28,36^ and declines in abundance and breeding success of forage-fish-dependent salmon, groundfish, birds, and mammals (Fig. 4). Evidence points to potential top-down and bottom-up mechanisms causing mid-trophic level forage reductions during the PMH^28,37^. For example, contrary to expectations, cold water zooplankton species that typically decline in abundance during warm water periods^25,38,39^ showed a weak negative or neutral to positive response (Fig. 3). The increase in zooplankton biomass overall, however, was dominated by smaller, warm water species, which likely hindered energy acquisition of zooplanktivores. The overall high biomass of warm and cold copepod species during the marine heatwave could also suggest possible reduced grazing pressure by forage fishes and other zooplanktivores due to greater consumption of those species by ectothermic predators with increased energetic demands during warm water conditions^28,37^. Declines in euphausiids, especially cool water associated species of *Thysanoessa inermis* and *T*. *longipes,* however, could have also limited energy transfer to upper trophic levels^40^. Overall, over half of our 187 time series showed a significant, multi-year response during the PMH; and intertidal to pelagic community metrics showed how prior disparate trends coalesced during the PMH. These results signify the magnitude of the effect of the PMH and suggest the GOA ecosystem had passed a tipping point that could no longer be buffered by the functional redundancy supporting resiliency in this system^41^.

The PMH affected biological metrics across the northern GOA, but we did observe some regional differences including some stronger responses in areas east (upstream) versus west (downstream) of Cook Inlet. These areas differ hydrographically in terms of mixing regimes affecting temperature and overall productivity, with Kodiak Island area west of Cook Inlet providing thermal refuge for some cold water-associated species (e.g., capelin)^42^.

### Human dimension

Changes in the GOA ecosystem during the PMH that affected groundfish and salmon stocks subsequently affected commercial fisheries and local communities. Like all marine predators, commercial fishers are constantly adapting to changes in the abundance and distribution of their target species^43,44^. While the abundance of some commercial species with lower latitude distributions (e.g., southern rock sole and juvenile sablefish) exhibited a positive response during the PMH, other species optimized to grow and survive at colder temperatures, such as cod, showed strongly negative effects^45,46^ (Fig. 4). Changes in groundfish abundance were accompanied by changes in distribution^47^ that can collectively affect overall fishery revenue ^48^. Resilience exists in fisheries and local economies through adjustments in the supply chain (e.g., price, timing of product delivery) and fisheries diversification^49^ so that individuals, communities, and commercial markets can adapt to heatwaves (Fig. 6). However, commercial species most negatively affected by the PMH in the GOA were among the highest revenue-producing fisheries representing a substantial portion of total earnings for vessels in the region. Additionally, uncertainty in recovery times associated with repeated fishery closures, rapid rates of change in target species, and damage to natural attractions (e.g., whale watching) created stress for fishing- and tourist-dependent communities^50,51^.

### Signals of recovery

It is difficult to predict when or whether the GOA ecosystem will return to a pre-PMH state. Examples from the 2011 Western Australian marine heatwave indicated that after 7 years, only parts of the ecosystem were showing significant signs of recovery^52^; whereas Chandrapavan et al. (2019)^53^ reported partial recovery of a crab stock only 18 months after a marine heatwave in Australia—with the recovery linked to the return of mean summer water temperatures to below 24 °C. Key factors in that system that influenced recovery rate for individual species included: (1) species at the latitudinal (thermal) limit of their range; (2) spatial overlap between species distribution and the extent of the warming event; (3) the stage of the life cycle that was affected; (4) life-cycle duration and maturation time of the species affected; and (5) management intervention^52^. Similar factors were at play during the PMH and partly explain short- versus long-term responses for some metrics. The magnitude of GOA heatwave, however, resulted in broad, cross-sectional effects and lack of return to a pre-heatwave state across multiple trophic levels.

Indications of return to pre-PMH levels by 2018 for time series with significant heatwave responses were strongest in metrics where shorter-term response times might be expected. However, even rocky intertidal macroalgal (*Fucus*) cover (Fig. 3) and whale encounter rates (Figs. 4, 6) showed some, albeit relatively minor, progress toward pre-PMH levels. Nonetheless, the GOA community through 2018 still appeared distinct from pre-PMH years (Fig. 5, Fig. S3). While additional prey and predator metrics were showing some indication in 2019 of trending back toward pre-PMH levels^33^, the re-intensification of the warming through fall 2018 and summer 2019^23,32^ and the return of some lower trophic level metrics (e.g., spring chlorophyll size composition, abundance of warm water copepods) to heatwave levels in 2019 (Batten, Campbell, Holderied, Hopcroft, Kimmel, McKinstry, Strom unpubl. data), suggest caution when interpreting observations during two years of an apparent heatwave hiatus.

### Future Gulf of Alaska

Recent warming events that began with the PMH are affecting most of Alaska, including the GOA, Bering Sea and Arctic Ocean, with recent years among the most anomalous on record^54^. Annual sea surface temperatures in the GOA and Bering Sea through 2019 showed that half or more of the warmest 10 of the past 119 years occurred after the onset of the PMH in 2014 (i.e., all years hence, with the exception of 2017 in the GOA; University of Alaska Fairbanks, Alaska Center for Climate Assessment and Policy, ACCAP). The sustained warm water anomalies over winter that were characteristic of the PMH were of particular concern for the GOA subarctic ecosystem. Cool winter temperatures can be important to slow metabolic demands for food and maintain lipid reserves for fish^55^. Bioenergetic winter stress was also hypothesized to contribute to increased mortality of adult Pacific cod in the GOA^37^. While some species and ontogenetic stages may initially respond favorably to warm periods in the GOA, thresholds exist in positive seasonal temperature-recruitment relationships^56^. Such changes do not appear to be gradual. For example, in the neighboring Bering Sea ecosystem, the absence of sea ice over a single winter resulted in a poleward shift in the zooplankton and fish community composition the following summer^57^.

Extreme climate events such as the PMH are an emerging driver of marine ecosystem dynamics with long-term impacts potentially greater than those of slower warming that leads to gradual reorganization and possible evolution and adaptation^58^. Models are successful in forecasting some physical aspects of events such as the PMH^59^; however, we are still searching for mechanisms to forecast biological change in these complex ecosystems^60–62^. Whereas our analyses did not identify mechanisms of biological change, our results do provide a foundation on which to develop hypotheses and test mechanistic links to physical drivers of change for specific taxa, life stages, trophic levels, and thermal niches. We expect that subsequent targeted analyses will identify unifying mechanisms of change to inform ecosystem models. Long-term monitoring efforts—coupled with targeted process studies—are critically important and contribute disproportionately to this effort^63^, such that informed management decisions are compromised without dedicated long-term monitoring programs^64^. Effective communication of knowledge regarding observed and projected change is also important, not only for maintaining support for long-term monitoring programs^65^, but also to evaluate trade-offs associated with climate change adaptation and effective management of marine resources during periods of rapid climate change or extreme climate events^66^.

## Methods

Biological time series (n = 187) within our northern GOA study area (Fig. 2) were obtained from several long-term research and monitoring programs in the region (Tables 1, S1). A single time series represented an annual measurement or mean value spanning at least 6 years from a single location or region. Time series were neither randomly selected nor consistent in spatial extent, yet they broadly represented examples of how lower to upper trophic level ecosystem components responded during the PMH in the northern GOA. Furthermore, only spatially replicated metrics were used to address potentially different responses in the eastern versus western study area (Fig. 2, Table S1).

Time series varied in length of 6 to 49 years between 1971 and 2019 (Table S1). For our primary analyses to assess GOA ecosystem response to the 2014–2016 PMH, we used all 187 time series from a 9-year period (2010–2018), providing the most years pre-PMH while minimizing missing years in time series (n = 8–9 years [91% of time series], n = 5–7 years [9% of time series]). Missing values during the 2010–2018 period were due to either more recent start of a time series or alternate year sampling; however, all time series were initiated by 2012 and included years post PMH. Additional analyses were conducted on a reduced number of time series that spanned longer time periods, 19–49 years, to assess biological trends during the 2014–2016 PMH relative to events of prior decades. Below we describe analytical approaches and general sample collection methods; see Table S1 for duration and frequency of sampling for each time series.

### Data treatment and statistical analyses

Annual arithmetic means were generated for all time series from (1) seasonal or monthly means, if multiple sampling events occurred throughout the year, or (2) individual samples, if sampling occurred during a single sampling period lasting shorter than a month. Metrics with skewed distributions of values varying by generally more than one order of magnitude were log transformed (log_10_(x + 1)) prior to calculating annual (geometric) means and annual means were back transformed prior to analysis. Statistically significant linear trends in seven annual time series (abundance of Steller sea lions, *Eumetopias jubatus,* and black-legged kittiwakes, *Rissa tridactyla*) were removed to reduce potentially confounding patterns that could mask a PMH response. In these few cases, residual values were used in the final analyses. Data were processed using Matlab R2017b (The MathWorks, Natick, MA) and R v3.6.3^67^.

We used dynamic factor analysis (DFA) to identify the number and shape of trends that best described common patterns in all time series^68^. Dynamic factor analyses were conducted in R using the multivariate autoregressive state-space modeling package^69^. We fit nine models that included three variance structures and up to three trends to the full set of 187 time series over a 9-year period that encompassed the PMH. Model variance structures that we tested included: (1) same variance and covariance (R matrix specified as ‘equalvarcov’); (2) same variance and no covariance (diagonal and equal); and (3) different variance and no covariance (diagonal and unequal). We restricted the analysis to identifying a maximum of three possible trends that might include a single response (positive or negative), multiple responses (e.g., change, then return to pre-PMH), and neutral or change not associated with the timing of PMH. We applied a z-score standardization to all time series prior to analysis and followed routine methods of fitting models outlined in Holmes et al. (2018)^70^. A time series was considered associated with the common trend if factor loadings were > 0.20 (absolute value)^71^.

Whereas DFA identifies common trends sequentially across years, community analysis identifies which individual years, regardless of numeric sequence, are most similar or dissimilar in their 187 biological metrics. We conducted two community analyses, hierarchical cluster analysis and nMDS using PRIMER v7 (PRIMER-e ltd, Quest Research Limited). The broad diversity in units and range of values in our various metrics dictated the use of normalized data (similar z-score noted above) and a resemblance matrix based on Euclidean distance, rather than square root transformation and Bray–Curtis similarity index more typically used for analysis of biological data with a common unit of measure^72,73^. Stress for nMDS analysis was considered acceptable if < 0.20. We used a similarity profile routine in cluster analysis (999 permutations, α = 0.05) and analysis of similarities in nMDS to test for significant differences among groups^72,74^. We also evaluated correlation of community metrics with ordination axes to compare responses of metric types with results of DFA.

### Sample collection and processing

All field sampling was conducted under Alaska state and U.S. federal permits issued to participating organizations and the institutional animal care and use committee of the University of Alaska Southeast for the humpback whale study. Data from commercial fisheries were collected and presented in accordance with confidentiality requirements of the Magnuson-Steven’s Fisheries Conservation and Management Act. No live animal experiments were conducted during this study.

### Phytoplankton

Satellite-derived chlorophyll concentration data from the Aqua MODIS sensor were downloaded from the NASA ocean color web portal (https://oceancolor.gsfc.nasa.gov/l3/). All satellite products were level-3, 4 km resolution, 8-day composites spanning 29–30 March to 8–9 October for all years, 2003–2018. We extracted all data within rectangular boxes drawn around the Seward and Kodiak sampling lines (Fig. 2). Each box was 74 km wide, and extended across all vessel-based oceanographic sampling stations (see below), with the Kodiak box extending an additional 56 km offshore to ensure that commonly occurring, transient mesoscale eddies were included. Each box was also split into shelf and oceanic zones. The boxes contain 1615–1698 pixels each, with shelf and oceanic boxes containing 665–1033 pixels. We excluded images with less than 30% coverage, such that all mean satellite-derived chlorophyll data included a minimum of 200 data points. Metrics used were chlorophyll biomass in each zone, and day of the year exhibiting peak chlorophyll biomass (shelf zones only).

In situ measures of the size composition of chlorophyll-containing particles were integrated at 10 m intervals from the upper 50 m at stations GAK1-13 along the Seward Line. Water samples were filtered using a 20 µm pore-size filter over 0.7 µm effective pore-size filter. Pigments were extracted in 90% acetone and chlorophyll content determined by fluorometry on board the ship. The metric used for this analysis was the fraction of total chlorophyll in particles > 20 µm (> 20 µm chl/total chl). The data were summarized by continental shelf (GAK1-9) and slope (GAK10-13) domains. For detailed methods see ref.^75^.

### Zooplankton and Ichthyoplankton

Microzooplankton samples were collected from 10 m depth at the chlorophyll collection stations (see above). Microzooplankton biomass was determined from settled water samples analyzed using inverted microscopy. Only cells ≥ 15 µm in longest dimension were included; biomass was determined from biovolumes using taxon- and fixative-specific conversion factors. Metrics used were microzooplankton biomass and microzooplankton community composition (fraction of biomass that was ciliates). Continental shelf and slope domains were differentiated as described for chlorophyll. For detailed methods see ref.^76^.

Zooplankton samples were collected using several sampling platforms. Continuous plankton recorders towed by commercial ships transiting through the study area to ports in Alaska and Asia collected samples throughout continental shelf and oceanic waters of the GOA (Fig. 2; for more details see ref.^25^). Research vessel-based net sampling was used to collect zooplankton samples along Line 8 in Shelikof Strait, Seward Line, in Prince William Sound (PWS), and Kachemak Bay (Fig. 2). Samples were collected during spring through fall using paired bongo nets, CalVet nets, or Multinets with 153, 202, 333, or 505 μm mesh. Nets were towed to a depth of 200 m maximum (or 5–10 m above the bottom when shallower) in the GOA and to 50 m maximum (or near bottom when shallower) in Prince William Sound and Kachemak Bay (for detailed methods see ref.^39,77,78^). For multi-decade time series that used various mesh sizes over time, we included species and life history stages that were less effected by differences in mesh size^78^. Metrics for zooplankton used in our analysis included overall zooplankton abundance and size^77^, copepod community size index^79^, and the abundance of warm and cool water associated copepods; with warm species distinguished as *Calanus pacificus*, *Clausocalanus* spp., *Corycaeus anglicus*, *Ctenocalanus* spp., *Mesocalanus tenuicornis*, and *Paracalanus parvus;* and cool species as *Calanus marshallae*, *Pseudocalanus* spp., *Arcartia longiremis*, *Oithona similis*, *Neocalanus cristatus*, *N. flemingeri*, *N. plumchrus*, and *Eucalanus bungii*^38^.

Large medusa jellies were collected during annual bottom trawl surveys of continental shelf waters in the western study area around Kodiak Island^80^. The catches for each year were scaled to the largest catch over the time series (which was arbitrarily scaled to a value of 100). Although jellies are incidental to targeted groundfish catch in the trawl, the data do provide a relative annual index of occurrence.

Larval fishes were collected during bongo net tows described above aboard research vessels in the western portion of our study area (Fig. 2) during spring using methods described by Matarese et al. (2003)^81^ and Laurel and Rogers (2020)^46^. We included the larval abundance of four numerically dominant groundfish species (walleye pollock *Gadus chalcogrammus*, Pacific cod *Gadus macrocephalus*, northern rock sole *Lepidopsetta polyxystra*, southern rock sole *L*. *bilineata*) and one forage fish species (Pacific sand lance *Ammodytes personatus*).

### Intertidal organisms

Rocky intertidal sampling was conducted at 21 sites in four regions across the northern GOA; including Prince William Sound, Kenai Peninsula (Kenai Fjords National Park), Kachemak Bay, and the Alaska Peninsula (Katmai National Park and Preserve). We selected a subsample of metrics representing a primary producer (macroalga *Fucus distichus*), predators (sea stars, *Dermasterias imbricata, Evasterias troschelii, Pisaster ochraceus, and Pycnopodia helianthoides*), and invertebrate prey (Pacific blue mussel, *Mytilus trossulus*). See ref.^82–84^ for detailed methods and analysis of rocky and mussel bed intertidal community changes during the PMH. We used percent cover of *Fucus* (individuals m^-2^ quadrat), counts of sea stars along a 50 m × 4 m transect (individuals 200 m^-2^) at rocky intertidal sites, and density of large (> 20 mm) mussels (individuals m^-2^ quadrat) at mussel bed sites. To standardize metrics among regions, *Fucus* was converted to normalized anomalies of percent cover, whereas raw counts were retained for sea stars and mussels. Additional indices of intertidal invertebrate availability to consumers included percent biomass of mussels and chitons (*Neoloricata* spp.) in glaucous-winged gull (*Larus glaucescens*) diets from Chowiet Island^85^.

### Forage fishes

We included juveniles and adults of three forage fishes (Pacific sand lance, Pacific capelin *Mallotus catervarius*, Pacific herring *Clupea pallasii*) that, when available, support high productivity and abundance of marine bird and mammal predators within our northern GOA study area^7,86–89^. The relative availability of sand lance and capelin to predators was assessed by the biomass in diets of black-legged kittiwakes (*Rissa tridactyla*), a surface forager, and rhinoceros auklets (*Cerorhinca monocerata*), a diving forager, on Middleton Island that sample forage fishes from throughout much of our eastern study area (Fig. 2) and from Chowiet Island in the western study area. Diet samples were collected during May-September^33,85^. Additional information on forage fishes included percent biomass of hexagrammids and fish (overall) from glaucous-winged gull diets on Chowiet Island.

We also included sand lance whole body energy content. Sand lance were collected using a purse seine, beach seine, herring jig, cast net, dip net, and gill net, with whole-body energy determined following von Biela et al. (2019)^34^. Samples were collected from PWS during July when annual body condition and lipid accumulation were highest^90^.

We used cumulative annual miles of milt from spawning adults in spring to represent herring abundance in PWS^91^ and third-year scale growth as an annual herring performance/condition index^92,93^.

### Groundfish

In addition to larval groundfish abundance described above, we also included metrics for five species of juvenile and adult groundfish. The abundances of juvenile (age-0) walleye pollock, Pacific cod, and saffron cod (*Eleginus gracilis*) were estimated from 17 beach seine sites in two bays on eastern Kodiak Island (Fig. 2). We measured juvenile (age-0) sablefish (*Anoplopoma fimbria*) growth from samples brought back to Middleton Island by nesting rhinoceros auklets, which forage throughout much of our eastern study area. The annual growth index represents changes in size of juvenile sablefish from June to August of each year. We used stock assessment model output for estimates of female spawning biomass for walleye pollock^94^, Pacific cod^95^, arrowtooth flounder (*Atheresthes stomias*)^96^, and sablefish^97^.

### Marine birds

Marine bird colony metrics included abundance of breeding pairs of black-legged kittiwakes at 44 colony sites from throughout PWS and sub-plots within colonies at Chowiet Island and common murres (*Uria aalge*) at Chowiet and East Amatuli Islands (Fig. 2), representing colonies in the eastern and western study areas. Reproductive success (chicks fledged pair^-1^) was also included for kittiwakes at all of the same colonies, rhinoceros auklets from Middleton and Chowiet Islands, and common murres at Chowiet and East Amatuli Islands. Additional reproductive success data were available from Chowiet Island only and included parakeet auklets (*Aethia psittacula*), tufted puffins (*Fratercula cirrhata*), glaucous-winged gulls, and pelagic cormorants (*Phalacrocorax pelagicus*). Annual estimates of kittiwake nesting abundance was summed for all colonies in PWS and annual reproductive success was first calculated for each colony as a whole, then averaged among colonies in PWS and among nest sites at the Middleton, Chowiet, and East Amatuli Island colonies (for more details see ref.^7,85,86,98^).

Marine bird abundance at sea was determined using strip transect surveys from aboard vessels conducting alongshore transects adjacent to the Alaska Peninsula and Kenai Peninsula intertidal study sites described above, from transects within PWS, and along the Seward Line between oceanographic sampling stations and transits within the region depicted in Fig. 2. We included time series of summer marine bird abundance for five species from alongshore transects adjacent to the intertidal sites: black oystercatcher (*Haematopus bachmani*), *Brachyramphus* murrelets (marbled murrelet, *B. marmoratus*, Kittlitz’s *murrelet B. brevirostris*), common murre, pigeon guillemot (*Cepphus columba*), and harlequin duck (*Histrionicus histrionicus*). Two time series each were used for fall (November and December) and winter (February and March) abundance of common murres and *Brachyramphus* murrelets in PWS. For Seward Line bird surveys in the GOA, we included time series from six species in three oceanographic domains (inner continental shelf, middle shelf, and oceanic; see Fig. 1 for boundaries); and two seasons, spring (May) and fall (September). Not all possible species-season-domain combinations were included; instead, we selected the season-domain combinations from periods when the species were most abundant. We included the species that best represented the different domains and foraging guilds, which included common murre (shelf associated diving piscivore), black-legged kittiwake (shelf associated surface feeding piscivore), sooty shearwater (*Ardenna grisea*; oceanic and outer shelf associated diving planktivore/ominvore), black-footed albatross (*Phoebastria albatrus*; outer shelf-oceanic associated surface feeding piscivore), northern fulmar (*Fulmarus glacialis*; outer shelf-oceanic associated surface feeding omnivore), and fork-tailed storm-petrel (*Oceanodroma furcata*; outer shelf-oceanic associated surface feeding and primarily planktivore).

All data were summarized to the same density metric of number km^-2^. For more details on specific methods see ref.^99,100^ for alongshore transects adjacent to intertidal sampling sites, ref.^101^ for PWS surveys, and ref.^102^ for Seward Line surveys.

### Marine mammals

Sea otter (*Enhydra lutris*) energy recovery rate during foraging was obtained at three of the four intertidal sampling regions; Alaska Peninsula, Kenai Peninsula, and PWS. Annual energy recovery rate for each region was obtained from direct observations of foraging bouts of individual otters during summer daylight hours and is a product of foraging dive length, interval between dives, proportion of dives where food is obtained, and energy density of prey (reported as kcal min^-1^). For more detailed methods see ref.^103^.

Pinniped abundance time series were generated using multiple methods. Alongshore, vessel-based surveys for Steller sea lions and harbor seals (*Phoca vitulina*) were conducted adjacent to intertidal sampling sites on the Alaska Peninsula and Kenai Peninsula. Densities were calculated as individuals km^-2^ from strip transect surveys^99^. Annual abundance of Steller sea lion pups and older age classes (hereafter “non-pups”) were also obtained from Chiswell Island, a single index site off the Kenai Peninsula in the eastern half of our study area. Remote cameras placed on this rookery were used to count pups and non-pups throughout the breeding season^104^. A third, population-wide metric of Steller sea lion abundance was generated from aerial surveys of all haul-out sites and rookeries (including Chiswell Island) throughout our study area (Fig. 2). Direct counts were conducted via observers in fixed-wing aircraft during summer. A custom-built model (agTrend) was used to estimate the abundance of sea lions ashore based on raw counts from aerial surveys and accounting for missed survey sites due to inclement weather, etc.^105^.

Surveys of humpback whale (*Megaptera novaeangliae*) in PWS were conducted during fall when whales are targeting prey populations, especially herring, prior to their southbound migration to low latitude breeding and calving grounds. Observers aboard vessels counted and individually identified whales based on fluke photographic identification to generate an encounter rate metric of number of unique whales per day^106^.

Time series of metrics for killer whales (*Orcinus orca*) included encounter rate, and number of animals per encounter. We used data from vessel-based surveys conducted during September and October in Montague Straight and lower Knight Island Passage, the western region and near the entrance to PWS^107^.

### Commercial harvest

We used salmon and groundfish harvest metrics from throughout both regions in our study area. We used salmon commercial harvest metrics (mass in lbs) compiled by the Alaska Department of Fish and Game^108^ for wild and hatchery-reared Chinook (*Oncorhynchus tshawytscha*), coho (*O. kisutch*), pink (*O. gorbuscha)* and chum salmon (*O. keta)* from Kodiak Island, in the western portion of our study area, and PWS-Copper River, in the eastern portion. Additionally, we obtained annual catch (lbs) and revenue (US$) for sockeye salmon (*O. nerka)*, sablefish, Pacific cod, and walleye pollock from six communities throughout our study area: Cordova, Valdez, Seward, Kenai, Homer, and Kodiak (Fig. 2). The data were from fish tickets submitted to the Alaska Commercial Fisheries Entry Commission (https://www.cfec.state.ak.us/), with post-season adjustments to landings. Landings by community were determined by the declared residency of the fishing permit holder. All revenues were set to 2018 dollars to adjust for inflation. Fishery-specific data from communities with fewer than four participants were excluded to maintain confidentiality.

## Supplementary Information

Supplementary files 1.

Supplementary Information 2.

Supplementary Information 3.

Supplementary Information 4.

## Data Availability

Data from Gulf Watch Alaska, Herring Research and Monitoring, and other *Exxon Valdez* Oil Spill Trustee Council supported programs are available at the Alaska Ocean Observing System Gulf of Alaska Data Portal (https://portal.aoos.org/gulf-of-alaska), which includes links to DataONE (dataone.org) for those datasets with DOIs. Data contributed by the U.S. Geological Survey are also available at https://doi.org/10.5066/F74J0C9Z and https://doi.org/10.5066/F7416V6H. All time series used in our analyses are also included in a comma-separated text  file in the supplementary materials. The Alaska Department of Fish & Game (ADF&G) retains intellectual property rights to data collected by or for ADF&G. Any dissemination of the data must credit ADF&G as the source, with a disclaimer that exonerates the department for errors or deficiencies in reproduction, subsequent analysis, or interpretation.
