# Supplementary material for: Ecosystem response persists after a prolonged marine heatwave
Source: Sci Rep. 2021 Mar 18;11:6235. doi: 10.1038/s41598-021-83818-5 (PMC7973763; doi:10.1038/s41598-021-83818-5)
Supplement: Supplementary file 1 — Supplementary files 1. [file 41598_2021_83818_MOESM1_ESM.docx]

# Ecosystem response persists after a prolonged marine heatwave

Robert M. Suryan^1*^, Mayumi L. Arimitsu^2^, Heather A. Coletti^3^, Russell R. Hopcroft^4^, Mandy R. Lindeberg^1^, Steven J. Barbeaux^5^, Sonia D. Batten^6^, William J. Burt^4^, Mary A. Bishop^7^, James L. Bodkin^8^, Richard Brenner^9^, Robert W. Campbell^7^, Daniel A. Cushing^10^, Seth L. Danielson^4^, Martin W. Dorn^5^, Brie Drummond^11^, Daniel Esler^8^, Thomas Gelatt^5^, Dana H. Hanselman^1^, Scott A. Hatch^12^, Stormy Haught^13^, Kris Holderied^14^, Katrin Iken^4^, David B. Irons^15^, Arthur B. Kettle^11^, David G. Kimmel^5^, Brenda Konar^4^, Kathy J. Kuletz^15^, Benjamin J. Laurel^16^, John M. Maniscalco^17^, Craig Matkin^18^, Caitlin A.E. McKinstry^7^, Daniel H. Monson^8^, John R. Moran^1^, Dan Olsen^18^, Wayne A. Palsson^5^, W. Scott Pegau^7^, John F. Piatt^8^, Lauren A. Rogers^5^, Nora A. Rojek^11^, Anne Schaefer^7^, Ingrid B. Spies^5^, Janice M. Straley^19^, Suzanne L. Strom^20^, Kathryn L. Sweeney^5^, Marysia Szymkowiak^1^, Benjamin P. Weitzman^14^, Ellen M. Yasumiishi^1^, Stephani G. Zador^5^

^1^Alaska Fisheries Science Center, National Oceanic and Atmospheric Administration, Juneau, Alaska, USA *corresponding author: [rob.suryan@noaa.gov](mailto:rob.suryan@noaa.gov)

^2^U.S. Geological Survey, Alaska Science Center, Juneau, Alaska, USA

^3^National Park Service, Fairbanks, Alaska, USA

^4^University of Alaska Fairbanks, Fairbanks, Alaska, USA

^5^Alaska Fisheries Science Center, National Oceanic and Atmospheric Administration, Seattle, Washington, USA

^6^Marine Biological Association, Nanaimo, BC, Canada, USA

^7^Prince William Sound Science Center, Cordova, Alaska, USA

^8^U.S. Geological Survey, Alaska Science Center, Anchorage, Alaska, USA

^9^Alaska Department of Fish and Game, Juneau, Alaska, USA

^10^Pole Star Ecological Research LLC, Anchorage, Alaska, USA

^11^U.S. Fish and Wildlife Service, Homer, Alaska, USA

^12^Institute for Seabird Research and Conservation, Anchorage, Alaska, USA

^13^Alaska Department of Fish and Game, Cordova, Alaska, USA

^14^National Ocean Service, National Oceanic and Atmospheric Administration, Homer, Alaska, USA

^15^U.S. Fish and Wildlife Service, Anchorage, Alaska, USA

^16^Alaska Fisheries Science Center, National Oceanic and Atmospheric Administration, Newport, Oregon, USA

^17^Alaska SeaLife Center, Seward, Alaska, USA

^18^North Gulf Oceanic Society, Homer, Alaska, USA

^19^University of Alaska Southeast, Sitka, Alaska, USA

^20^Shannon Point Marine Center, Western Washington University, Anacortes, Washington, USA

## Supporting Information

Table S1. Biological time series (n = 187) used to assess ecosystem response to a multi-year marine heatwave in the Gulf of Alaska (GOA). Time series include unique identification (ID) number to link each time series with supplementary data file provided, taxonomic group, sampling method, geographic domain where sampled, east or west (or both) region of study area (Fig. 2), derived metric, time series start year, end year, and number of years (n; most, but not all are sampled annually), factor loadings from dynamic factor analysis, and data contributor. Note that a positive factor loading indicates the time series relates positively to the common trend (the common trend is a negative response during the PMH [Fig. 3], a positive factor loading refers to a negative response during the PMH) and vice versa. We use a factor loading of > 0.20 (absolute value) as the cutoff for whether or not a time series significantly relates to the common trend. Data contributors were Gulf Watch Alaska (GWA), Herring Research and Monitoring Program (HRM), Alaska Fisheries Science Center (AFSC), Alaska Department of Fish and Game (ADFG), U.S. Fish and Wildlife Service (USFWS), Alaska SeaLife Center (ASLC), and Northern Gulf of Alaska Long-Term Ecological Research (NGA LTER). See Methods for details on derived metrics and additional supplementary data files described below.

| ID | Taxa | Method | Domain | GOA region | Derived metric | Start year | End year | n (years) | Factor loadings | Data contributor |
| --- | --- | --- | --- | --- | --- | --- | --- | --- | --- | --- |
| 1 | Phytoplankton | Satellite | Seward Line Shelf | east | Chlorophyll biomass | 2003 | 2018 | 16 | 0.222 | NGA LTER |
| 2 | Phytoplankton | Satellite | Seward Line Slope | east | Chlorophyll biomass | 2003 | 2018 | 16 | 0.206 | NGA LTER |
| 3 | Phytoplankton | Satellite | Seward Line Shelf | east | Chlorophyll peak day | 2003 | 2018 | 15 | 0.107 | NGA LTER |
| 4 | Phytoplankton | Satellite | Kodiak Line Shelf | west | Chlorophyll biomass | 2003 | 2018 | 16 | 0.076 | NGA LTER |
| 5 | Phytoplankton | Satellite | Kodiak Line Slope | west | Chlorophyll biomass | 2003 | 2018 | 16 | 0.223 | NGA LTER |
| 6 | Phytoplankton | Satellite | Kodiak Line Shelf | west | Chlorophyll peak day | 2003 | 2018 | 13 | 0.241 | NGA LTER |
| 7 | Phytoplankton | Water sample from CTD | Seward Line Shelf | east | Fraction chlorophyll >20 µm spring | 2011 | 2019 | 8 | -0.032 | NGA LTER |
| 8 | Phytoplankton | Water sample from CTD | Seward Line Slope | east | Fraction chlorophyll >20 µm spring | 2011 | 2019 | 8 | -0.216 | NGA LTER |
| 9 | Phytoplankton | Water sample from CTD | Seward Line Shelf | east | Fraction chlorophyll >20 µm fall | 2011 | 2019 | 8 | -0.300 | NGA LTER |
| 10 | Phytoplankton | Water sample from CTD | Seward Line Slope | east | Fraction chlorophyll >20 µm fall | 2011 | 2019 | 8 | -0.201 | NGA LTER |
| 11 | Zooplankton | Water sample from CTD | Seward Line Shelf | east | Microzooplankton biomass spring | 2011 | 2018 | 6 | 0.322 | NGA LTER |
| 12 | Zooplankton | Water sample from CTD | Seward Line Slope | east | Microzooplankton biomass spring | 2011 | 2018 | 6 | 0.309 | NGA LTER |
| 13 | Zooplankton | Water sample from CTD | Seward Line Shelf | east | Microzooplankton biomass fall | 2011 | 2018 | 7 | -0.278 | NGA LTER |
| 14 | Zooplankton | Water sample from CTD | Seward Line Slope | east | Microzooplankton biomass fall | 2013 | 2018 | 6 | -0.173 | NGA LTER |
| 15 | Zooplankton | Water sample from CTD | Seward Line Shelf | east | Microzooplankton fraction of ciliates spring | 2011 | 2018 | 6 | -0.073 | NGA LTER |
| 16 | Zooplankton | Water sample from CTD | Seward Line Slope | east | Microzooplankton fraction of ciliates spring | 2011 | 2018 | 6 | 0.142 | NGA LTER |
| 17 | Zooplankton | Water sample from CTD | Seward Line Shelf | east | Microzooplankton fraction of ciliates fall | 2011 | 2018 | 7 | 0.179 | NGA LTER |
| 18 | Zooplankton | Water sample from CTD | Seward Line Slope | east | Microzooplankton fraction of ciliates fall | 2013 | 2018 | 6 | 0.151 | NGA LTER |
| 19 | Zooplankton | Plankton net | Shelikof Strait - Line 8 | west | *Calanus pacificus* abundance | 1990 | 2017 | 25 | -0.241 | AFSC |
| 20 | Zooplankton | Plankton net | Shelikof Strait - Line 8 | west | *Calanus marshallae* abundance | 1990 | 2017 | 25 | -0.350 | AFSC |
| 21 | Zooplankton | Continuous plankton recorder | Shelf | east | Copepod Size Index | 2000 | 2018 | 19 | -0.136 | GWA |
| 22 | Zooplankton | Continuous plankton recorder | Oceanic | east | Copepod Size Index | 2000 | 2018 | 19 | 0.233 | GWA |
| 23 | Zooplankton | Continuous plankton recorder | Shelf | east | Warm water zooplankton abundance | 2000 | 2018 | 19 | -0.205 | GWA |
| 24 | Zooplankton | Continuous plankton recorder | Oceanic | east | Warm water zooplankton abundance | 2000 | 2018 | 19 | -0.271 | GWA |
| 25 | Zooplankton | Continuous plankton recorder | Shelf | east | Cool water zooplankton abundance | 2000 | 2018 | 19 | -0.215 | GWA |
| 26 | Zooplankton | Continuous plankton recorder | Oceanic | east | Cool water zooplankton abundance | 2000 | 2018 | 19 | -0.170 | GWA |
| 27 | Zooplankton | Plankton net | Seward Line Shelf | east | Spring zooplankton size | 1998 | 2019 | 22 | -0.254 | GWA |
| 28 | Zooplankton | Plankton net | Seward Line Shelf | east | Fall zooplankton size | 2005 | 2018 | 14 | 0.242 | GWA |
| 29 | Zooplankton | Plankton net | Kachemak Bay | east | Copepod Size Index | 2012 | 2017 | 6 | -0.055 | GWA |
| 30 | Zooplankton | Plankton net | Kachemak Bay | east | All zooplankton abundance | 2012 | 2017 | 6 | -0.243 | GWA |
| 31 | Zooplankton | Plankton net | Kachemak Bay | east | Warm water zooplankton abundance | 2012 | 2017 | 6 | -0.385 | GWA |
| 32 | Zooplankton | Plankton net | Kachemak Bay | east | Cool water zooplankton abundance | 2012 | 2017 | 6 | -0.106 | GWA |
| 33 | Zooplankton | Plankton net | Prince William Sound | east | Copepod Size Index | 2010 | 2018 | 9 | -0.326 | GWA |
| 34 | Zooplankton | Plankton net | Prince William Sound | east | All zooplankton abundance | 2010 | 2018 | 9 | -0.370 | GWA |
| 35 | Zooplankton | Plankton net | Prince William Sound | east | Warm water zooplankton abundance | 2010 | 2018 | 9 | -0.303 | GWA |
| 36 | Zooplankton | Plankton net | Prince William Sound | east | Cool water zooplankton abundance | 2010 | 2018 | 9 | -0.054 | GWA |
| 37 | Zooplankton | Bottom trawl | Kodiak Region | west | Jellyfish medusae | 1984 | 2019 | 17 | 0.062 | AFSC |
| 38 | Intertidal organism | Quadrat | Prince William Sound | east | Pacific blue mussel density | 2010 | 2018 | 9 | -0.153 | GWA |
| 39 | Intertidal organism | Transect | Prince William Sound | east | Sea star density | 2010 | 2018 | 9 | 0.271 | GWA |
| 40 | Intertidal organism | Quadrat | Prince William Sound | east | *Fucus* percent cover | 2007 | 2018 | 10 | 0.318 | GWA |
| 41 | Intertidal organism | Quadrat | Kenai Peninsula | east | Pacific blue mussel density | 2008 | 2018 | 11 | -0.321 | GWA |
| 42 | Intertidal organism | Transect | Kenai Peninsula | east | Sea star density | 2008 | 2018 | 11 | 0.400 | GWA |
| 43 | Intertidal organism | Quadrat | Kenai Peninsula | east | *Fucus* percent cover | 2008 | 2018 | 11 | 0.363 | GWA |
| 44 | Intertidal organism | Quadrat | Kachemak Bay | east | Pacific blue mussel density | 2012 | 2018 | 7 | -0.162 | GWA |
| 45 | Intertidal organism | Transect | Kachemak Bay | east | Sea star density | 2009 | 2018 | 9 | -0.060 | GWA |
| 46 | Intertidal organism | Quadrat | Kachemak Bay | east | *Fucus* percent cover | 2012 | 2018 | 7 | 0.365 | GWA |
| 47 | Intertidal organism | Quadrat | Alaska Peninsula | west | Pacific blue mussel density | 2008 | 2018 | 10 | -0.301 | GWA |
| 48 | Intertidal organism | Transect | Alaska Peninsula | west | Sea star density | 2006 | 2018 | 11 | 0.376 | GWA |
| 49 | Intertidal organism | Quadrat | Alaska Peninsula | west | *Fucus* percent cover | 2006 | 2018 | 12 | 0.250 | GWA |
| 50 | Intertidal organism | Marine bird diet | Chowiet | west | Mytilus percent biomass in glaucous-winged gull diets | 2004 | 2019 | 15 | -0.239 | USFWS |
| 51 | Intertidal organism | Marine bird diet | Chowiet | west | Chiton percent biomass in glaucous-winged gull diets | 2004 | 2019 | 15 | 0.152 | USFWS |
| 52 | Forage Fish | Aerial survey | Prince William Sound | east | Pacific herring miles of spawn | 1974 | 2019 | 46 | 0.423 | HRM |
| 53 | Forage Fish | Scale growth increment | Prince William Sound | east | Pacific herring growth to age-4 | 1979 | 2017 | 39 | 0.380 | HRM |
| 54 | Forage Fish | Plankton net | Kodiak region | west | Sand lance larvae density | 1981 | 2017 | 32 | -0.295 | AFSC |
| 55 | Forage Fish | Marine bird diet | Middleton Island region | east | Sand lance percent biomass from black-legged kittiwake diet | 1978 | 2019 | 26 | -0.375 | GWA |
| 56 | Forage Fish | Marine bird diet | Middleton Island region | east | Capelin percent biomass from black-legged kittiwake diet | 1978 | 2019 | 26 | 0.340 | GWA |
| 57 | Forage Fish | Marine bird diet | Middleton Island region | east | Sand lance percent biomass from rhinoceros auklet diet | 1978 | 2019 | 30 | -0.373 | GWA |
| 58 | Forage Fish | Marine bird diet | Middleton Island region | east | Capelin percent biomass from rhinoceros auklet diet | 1978 | 2019 | 30 | 0.345 | GWA |
| 59 | Forage Fish | Marine bird diet | Chowiet | west | Sand lance percent biomass from rhinoceros auklet diet | 1998 | 2019 | 16 | 0.131 | USFWS |
| 60 | Forage Fish | Marine bird diet | Chowiet | west | Capelin percent biomass from rhinoceros auklet diet | 1998 | 2019 | 16 | 0.006 | USFWS |
| 61 | Forage Fish | Marine bird diet | Chowiet | west | Hexagrammid percent biomass in glaucous-winged gull diets | 1998 | 2019 | 14 | -0.255 | USFWS |
| 62 | Forage Fish | Marine bird diet | Chowiet | west | Fish percent volume in glaucous-winged gull diets | 2004 | 2019 | 15 | -0.120 | USFWS |
| 63 | Forage Fish | nets (seine, dip, gill, cast), hook and line | Prince William Sound | east | Sand lance whole body energy content | 2012 | 2018 | 6 | 0.086 | GWA |
| 64 | Groundfish | Plankton net | Kodiak region | west | Walleye pollock larvae density | 1981 | 2017 | 32 | 0.122 | AFSC |
| 65 | Groundfish | Plankton net | Kodiak region | west | Pacific cod larvae density | 1981 | 2017 | 32 | 0.153 | AFSC |
| 66 | Groundfish | Plankton net | Kodiak region | west | Northern rock sole larvae density | 1981 | 2017 | 32 | 0.046 | AFSC |
| 67 | Groundfish | Plankton net | Kodiak region | west | Southern rock sole larvae density | 1981 | 2017 | 32 | -0.252 | AFSC |
| 68 | Groundfish | Marine bird diet | Middleton Island region | east | Sablefish juvenile growth index, increase in length of fish from June to August | 1990 | 2019 | 27 | -0.222 | GWA |
| 69 | Groundfish | Beach seine | Kodiak Island | west | Walleye pollock age-0 abundance | 2006 | 2018 | 13 | -0.339 | AFSC |
| 70 | Groundfish | Beach seine | Kodiak Island | west | Pacific cod age-0 abundance | 2006 | 2018 | 13 | -0.039 | AFSC |
| 71 | Groundfish | Beach seine | Kodiak Island | west | Saffron cod age-0 abundance | 2006 | 2018 | 13 | -0.143 | AFSC |
| 72 | Groundfish | Stock assessment model | All Alaskan waters | both | Sablefish female spawning stock biomass | 1971 | 2019 | 49 | 0.405 | AFSC |
| 73 | Groundfish | Stock assessment model | Gulf of Alaska | both | Walleye pollock female spawning stock biomass | 1971 | 2019 | 49 | 0.026 | AFSC |
| 74 | Groundfish | Stock assessment model | Gulf of Alaska | both | Pacific cod female spawning stock biomass | 1977 | 2019 | 43 | 0.412 | AFSC |
| 75 | Groundfish | Stock assessment model | Gulf of Alaska | both | Arrowtooth flounder female spawning stock biomass | 1977 | 2019 | 43 | 0.438 | AFSC |
| 76 | Marine bird | Land-based survey | Chowiet | west | Common murre breeding success | 1979 | 2019 | 25 | -0.153 | USFWS |
| 77 | Marine bird | Land-based survey | Chowiet | west | Common murre abundance | 1989 | 2019 | 22 | 0.322 | USFWS |
| 78 | Marine bird | Land-based survey | Chowiet | west | Parakeet auklet breeding success | 1998 | 2019 | 16 | -0.037 | USFWS |
| 79 | Marine bird | Land-based survey | Chowiet | west | Rhinoceros auklet breeding success | 2012 | 2019 | 7 | 0.022 | USFWS |
| 80 | Marine bird | Land-based survey | Chowiet | west | Tufted puffin breeding success | 2005 | 2019 | 14 | -0.074 | USFWS |
| 81 | Marine bird | Land-based survey | Chowiet | west | Black-legged kittiwake breeding success | 1979 | 2019 | 24 | -0.179 | USFWS |
| 82 | Marine bird | Land-based survey | Chowiet | west | Black-legged kittiwake abundance | 1977 | 2019 | 28 | 0.075 | USFWS |
| 83 | Marine bird | Land-based survey | Chowiet | west | Glaucous-winged gull breeding success | 1998 | 2019 | 12 | -0.203 | USFWS |
| 84 | Marine bird | Land-based survey | Chowiet | west | Pelagic cormorant breeding success | 2010 | 2019 | 8 | 0.053 | USFWS |
| 85 | Marine bird | Land-based survey | East Amatuli | west | Common murre breeding success | 1993 | 2018 | 21 | 0.294 | USFWS |
| 86 | Marine bird | Land-based survey | East Amatuli | west | Common murre abundance | 1993 | 2018 | 23 | 0.403 | USFWS |
| 87 | Marine bird | Land-based survey | East Amatuli | west | Black-legged kittiwake breeding success | 1993 | 2019 | 24 | 0.114 | USFWS |
| 88 | Marine bird | Land-based survey | East Amatuli | west | Fork tailed storm petrel breeding success | 1998 | 2019 | 20 | 0.170 | USFWS |
| 89 | Marine bird | Vessel-based survey | Prince William Sound | east | Black-legged kittiwake breeding success | 1985 | 2019 | 35 | 0.187 | USFWS |
| 90 | Marine bird | Vessel-based survey | Prince William Sound | east | Black-legged kittiwake nest abundance | 1985 | 2019 | 35 | 0.375 | USFWS |
| 91 | Marine bird | Land-based survey | Middleton Island | east | Black-legged kittiwake breeding success | 1978 | 2019 | 40 | 0.345 | GWA |
| 92 | Marine bird | Land-based survey | Middleton Island | east | Rhinoceros auklet breeding success | 1995 | 2019 | 23 | 0.024 | GWA |
| 93 | Marine bird | Vessel-based survey | Prince William Sound | east | Common murre density November-December | 2007 | 2017 | 11 | -0.024 | GWA |
| 94 | Marine bird | Vessel-based survey | Prince William Sound | east | Common murre density February-March | 2008 | 2018 | 11 | 0.054 | GWA |
| 95 | Marine bird | Vessel-based survey | Prince William Sound | east | Brachyramphus murrelet density November-December | 2007 | 2017 | 11 | 0.005 | GWA |
| 96 | Marine bird | Vessel-based survey | Prince William Sound | east | Brachyramphus murrelet density February-March | 2008 | 2018 | 11 | -0.305 | GWA |
| 97 | Marine bird | Vessel-based survey | Seward Line - inner shelf | east | Common murre abundance May | 2007 | 2019 | 12 | -0.039 | GWA |
| 98 | Marine bird | Vessel-based survey | Seward Line - inner shelf | east | Common murre abundance September | 2006 | 2019 | 13 | 0.083 | GWA |
| 99 | Marine bird | Vessel-based survey | Seward Line - inner shelf | east | Black-legged kittiwake abundance May | 2007 | 2019 | 12 | -0.189 | GWA |
| 100 | Marine bird | Vessel-based survey | Seward Line - inner shelf | east | Black-legged kittiwake abundance September | 2006 | 2019 | 13 | 0.223 | GWA |
| 101 | Marine bird | Vessel-based survey | Seward Line - inner shelf | east | Sooty shearwater abundance May | 2007 | 2019 | 12 | 0.080 | GWA |
| 102 | Marine bird | Vessel-based survey | Seward Line - oceanic | east | Sooty shearwater abundance May | 2007 | 2019 | 10 | -0.077 | GWA |
| 103 | Marine bird | Vessel-based survey | Seward Line - inner shelf | east | Sooty shearwater abundance September | 2006 | 2019 | 13 | 0.191 | GWA |
| 104 | Marine bird | Vessel-based survey | Seward Line - oceanic | east | Sooty shearwater abundance September | 2006 | 2019 | 13 | 0.141 | GWA |
| 105 | Marine bird | Vessel-based survey | Seward Line - middle shelf | east | Black-footed albatross abundance May | 2007 | 2019 | 12 | -0.038 | GWA |
| 106 | Marine bird | Vessel-based survey | Seward Line - oceanic | east | Black-footed albatross abundance May | 2007 | 2019 | 10 | 0.093 | GWA |
| 107 | Marine bird | Vessel-based survey | Seward Line - middle shelf | east | Black-footed albatross abundance September | 2006 | 2019 | 13 | 0.159 | GWA |
| 108 | Marine bird | Vessel-based survey | Seward Line - oceanic | east | Black-footed albatross abundance September | 2006 | 2019 | 13 | 0.105 | GWA |
| 109 | Marine bird | Vessel-based survey | Seward Line - middle shelf | east | Northern fulmar abundance May | 2007 | 2019 | 12 | -0.208 | GWA |
| 110 | Marine bird | Vessel-based survey | Seward Line - oceanic | east | Northern fulmar abundance May | 2007 | 2019 | 10 | -0.121 | GWA |
| 111 | Marine bird | Vessel-based survey | Seward Line - middle shelf | east | Northern fulmar abundance September | 2006 | 2019 | 13 | 0.004 | GWA |
| 112 | Marine bird | Vessel-based survey | Seward Line - oceanic | east | Northern fulmar abundance September | 2006 | 2019 | 13 | -0.076 | GWA |
| 113 | Marine bird | Vessel-based survey | Seward Line - inner shelf | east | Fork-tailed storm petrel abundance May | 2007 | 2019 | 12 | -0.335 | GWA |
| 114 | Marine bird | Vessel-based survey | Seward Line - oceanic | east | Fork-tailed storm petrel abundance May | 2007 | 2019 | 10 | 0.334 | GWA |
| 115 | Marine bird | Vessel-based survey | Seward Line - inner shelf | east | Fork-tailed storm petrel abundance September | 2006 | 2019 | 13 | -0.163 | GWA |
| 116 | Marine bird | Vessel-based survey | Seward Line - oceanic | east | Fork-tailed storm petrel abundance September | 2006 | 2019 | 13 | -0.006 | GWA |
| 117 | Marine bird | Vessel-based survey | Alaska Peninsula | west | Black oystercatcher abundance summer | 2006 | 2018 | 12 | -0.345 | GWA |
| 118 | Marine bird | Vessel-based survey | Alaska Peninsula | west | Brachyramphus murrelet abundance summer | 2006 | 2018 | 12 | 0.233 | GWA |
| 119 | Marine bird | Vessel-based survey | Alaska Peninsula | west | Common murre abundance summer | 2006 | 2018 | 12 | -0.006 | GWA |
| 120 | Marine bird | Vessel-based survey | Alaska Peninsula | west | Harlequin duck abundance summer | 2006 | 2018 | 12 | 0.136 | GWA |
| 121 | Marine bird | Vessel-based survey | Alaska Peninsula | west | Pigeon guillemot abundance summer | 2006 | 2018 | 12 | 0.225 | GWA |
| 122 | Marine bird | Vessel-based survey | Kenai Peninsula | east | Black oystercatcher abundance summer | 2007 | 2018 | 12 | -0.444 | GWA |
| 123 | Marine bird | Vessel-based survey | Kenai Peninsula | east | Brachyramphus murrelet abundance summer | 2007 | 2018 | 12 | 0.218 | GWA |
| 124 | Marine bird | Vessel-based survey | Kenai Peninsula | east | Common murre abundance summer | 2007 | 2018 | 12 | -0.249 | GWA |
| 125 | Marine bird | Vessel-based survey | Kenai Peninsula | east | Harlequin duck abundance summer | 2007 | 2018 | 12 | 0.047 | GWA |
| 126 | Marine bird | Vessel-based survey | Kenai Peninsula | east | Pigeon guillemot abundance summer | 2007 | 2018 | 12 | 0.262 | GWA |
| 127 | Marine bird | Marine bird diet | Chowiet | west | Birds percent volume in glaucous-winged gull diets | 2004 | 2019 | 15 | 0.157 | USFWS |
| 128 | Otter | Shore-based observation | Kenai Peninsula | east | Sea otter foraging (energy recovery rate) | 2007 | 2018 | 12 | 0.034 | GWA |
| 129 | Otter | Shore-based observation | Alaska Peninsula | west | Sea otter foraging (energy recovery rate) | 2006 | 2018 | 12 | 0.232 | GWA |
| 130 | Otter | Shore-based observation | Prince William Sound | east | Sea otter foraging (energy recovery rate) | 2003 | 2018 | 13 | -0.014 | GWA |
| 131 | Pinniped | Vessel-based survey | Alaska Peninsula | west | Harbor seal abundance in water and on land | 2006 | 2018 | 12 | -0.294 | GWA |
| 132 | Pinniped | Vessel-based survey | Kenai Peninsula | east | Harbor seal abundance in water and on land | 2007 | 2018 | 12 | 0.175 | GWA |
| 133 | Pinniped | Vessel-based survey | Alaska Peninsula | west | Steller sea lion abundance in water and on land | 2006 | 2018 | 12 | 0.162 | GWA |
| 134 | Pinniped | Vessel-based survey | Kenai Peninsula | east | Steller sea lion abundance in water and on land | 2007 | 2018 | 12 | -0.140 | GWA |
| 135 | Pinniped | Aerial survey | Western GOA | west | Steller sea lion non-pup abundance on haul outs and rookeries | 1978 | 2019 | 28 | -0.295 | AFSC |
| 136 | Pinniped | Aerial survey | Western GOA | west | Steller sea lion pup abundance on haul outs and rookeries | 1978 | 2019 | 28 | 0.298 | AFSC |
| 137 | Pinniped | Aerial survey | Eastern GOA | east | Steller sea lion non-pup abundance on haul outs and rookeries | 1978 | 2019 | 24 | 0.165 | AFSC |
| 138 | Pinniped | Aerial survey | Eastern GOA | east | Steller sea lion pup abundance on haul outs and rookeries | 1978 | 2019 | 22 | 0.412 | AFSC |
| 139 | Pinniped | Island - based remote camera observation | Chiswell Island | east | Steller sea lion age-1+ abundance on Chiswell Island | 2005 | 2019 | 15 | 0.231 | ASLC |
| 140 | Pinniped | Island - based remote camera observation | Chiswell Island | east | Steller sea lion pup abundance on Chiswell Island | 2005 | 2019 | 15 | 0.171 | ASLC |
| 141 | Whale | Vessel-based survey | Prince William Sound | east | Humpback whale individuals encountered per day of surveying | 2008 | 2019 | 8 | 0.270 | GWA |
| 142 | Whale | Vessel-based survey | Prince William Sound | east | Killer whale individual per day encounter rate | 2005 | 2019 | 15 | 0.191 | GWA |
| 143 | Whale | Vessel-based survey | Prince William Sound | east | Killer whale encounter rate | 2005 | 2019 | 15 | 0.128 | GWA |
| 144 | Salmon | Commercial fishing permit holders | Prince William Sound and Copper River | east | Chinook salmon, pounds harvested | 1971 | 2019 | 49 | 0.059 | ADFG |
| 145 | Salmon | Commercial fishing permit holders | Prince William Sound and Copper River | east | Coho salmon pounds harvested | 1971 | 2019 | 49 | -0.190 | ADFG |
| 146 | Salmon | Commercial fishing permit holders | Prince William Sound and Copper River | east | Pink salmon pounds harvested | 1971 | 2019 | 49 | 0.088 | ADFG |
| 147 | Salmon | Commercial fishing permit holders | Prince William Sound and Copper River | east | Chum salmon pounds harvested | 1971 | 2019 | 49 | -0.196 | ADFG |
| 148 | Salmon | Commercial fishing permit holders | Kodiak | west | Chinook salmon, pounds harvested | 1971 | 2019 | 49 | 0.285 | ADFG |
| 149 | Salmon | Commercial fishing permit holders | Kodiak | west | Coho salmon pounds harvested | 1971 | 2019 | 49 | -0.217 | ADFG |
| 150 | Salmon | Commercial fishing permit holders | Kodiak | west | Pink salmon pounds harvested | 1971 | 2019 | 49 | 0.002 | ADFG |
| 151 | Salmon | Commercial fishing permit holders | Kodiak | west | Chum salmon pounds harvested | 1971 | 2019 | 49 | -0.130 | ADFG |
| 152 | Salmon | Commercial fishing permit holders | Valdez | east | Sockeye salmon revenue | 1985 | 2018 | 34 | 0.209 | AFSC |
| 153 | Salmon | Commercial fishing permit holders | Valdez | east | Sockeye salmon pounds harvested | 1985 | 2018 | 34 | 0.223 | AFSC |
| 154 | Salmon | Commercial fishing permit holders | Seward | east | Sockeye salmon revenue | 1985 | 2018 | 34 | 0.149 | AFSC |
| 155 | Salmon | Commercial fishing permit holders | Seward | east | Sockeye salmon pounds harvested | 1985 | 2018 | 34 | 0.199 | AFSC |
| 156 | Salmon | Commercial fishing permit holders | Kodiak | west | Sockeye salmon revenue | 1985 | 2018 | 34 | 0.172 | AFSC |
| 157 | Salmon | Commercial fishing permit holders | Kodiak | west | Sockeye salmon pounds harvested | 1985 | 2018 | 34 | 0.098 | AFSC |
| 158 | Salmon | Commercial fishing permit holders | Kenai Peninsula | east | Sockeye salmon revenue | 1985 | 2018 | 34 | 0.282 | AFSC |
| 159 | Salmon | Commercial fishing permit holders | Kenai Peninsula | east | Sockeye salmon pounds harvested | 1985 | 2018 | 34 | 0.257 | AFSC |
| 160 | Salmon | Commercial fishing permit holders | Homer | east | Sockeye salmon revenue | 1985 | 2018 | 34 | 0.351 | AFSC |
| 161 | Salmon | Commercial fishing permit holders | Homer | east | Sockeye salmon pounds harvested | 1985 | 2018 | 34 | 0.302 | AFSC |
| 162 | Salmon | Commercial fishing permit holders | Cordova | east | Sockeye salmon revenue | 1985 | 2018 | 34 | 0.401 | AFSC |
| 163 | Salmon | Commercial fishing permit holders | Cordova | east | Sockeye salmon pounds harvested | 1985 | 2018 | 34 | 0.401 | AFSC |
| 164 | Groundfish | Commercial fishing permit holders | Valdez | east | Sablefish revenue | 1985 | 2018 | 34 | -0.015 | AFSC |
| 165 | Groundfish | Commercial fishing permit holders | Valdez | east | Sablefish pounds harvested | 1985 | 2018 | 34 | -0.019 | AFSC |
| 166 | Groundfish | Commercial fishing permit holders | Seward | east | Sablefish revenue | 1985 | 2018 | 34 | 0.166 | AFSC |
| 167 | Groundfish | Commercial fishing permit holders | Seward | east | Sablefish pounds harvested | 1985 | 2018 | 34 | 0.125 | AFSC |
| 168 | Groundfish | Commercial fishing permit holders | Seward | east | Pacific cod revenue | 1987 | 2018 | 31 | 0.246 | AFSC |
| 169 | Groundfish | Commercial fishing permit holders | Seward | east | Pacific cod pounds harvested | 1987 | 2018 | 31 | 0.312 | AFSC |
| 170 | Groundfish | Commercial fishing permit holders | Kodiak | west | Sablefish revenue | 1985 | 2018 | 34 | 0.239 | AFSC |
| 171 | Groundfish | Commercial fishing permit holders | Kodiak | west | Sablefish pounds harvested | 1985 | 2018 | 34 | 0.113 | AFSC |
| 172 | Groundfish | Commercial fishing permit holders | Kodiak | west | Pollock revenue | 1985 | 2018 | 34 | -0.099 | AFSC |
| 173 | Groundfish | Commercial fishing permit holders | Kodiak | west | Pollock pounds harvested | 1985 | 2018 | 34 | -0.390 | AFSC |
| 174 | Groundfish | Commercial fishing permit holders | Kodiak | west | Pacific cod revenue | 1985 | 2018 | 34 | 0.350 | AFSC |
| 175 | Groundfish | Commercial fishing permit holders | Kodiak | west | Pacific cod pounds harvested | 1985 | 2018 | 34 | 0.383 | AFSC |
| 176 | Groundfish | Commercial fishing permit holders | Kenai Peninsula | east | Pacific cod revenue | 1987 | 2018 | 29 | 0.275 | AFSC |
| 177 | Groundfish | Commercial fishing permit holders | Kenai Peninsula | east | Pacific cod pounds harvested | 1987 | 2018 | 29 | 0.285 | AFSC |
| 178 | Groundfish | Commercial fishing permit holders | Homer | east | Sablefish revenue | 1985 | 2018 | 34 | -0.281 | AFSC |
| 179 | Groundfish | Commercial fishing permit holders | Homer | east | Sablefish pounds harvested | 1985 | 2018 | 34 | -0.305 | AFSC |
| 180 | Groundfish | Commercial fishing permit holders | Homer | east | Pollock revenue | 1987 | 2018 | 27 | 0.390 | AFSC |
| 181 | Groundfish | Commercial fishing permit holders | Homer | east | Pollock pounds harvested | 1987 | 2018 | 27 | 0.389 | AFSC |
| 182 | Groundfish | Commercial fishing permit holders | Homer | east | Pacific cod revenue | 1985 | 2018 | 34 | 0.352 | AFSC |
| 183 | Groundfish | Commercial fishing permit holders | Homer | east | Pacific cod pounds harvested | 1985 | 2018 | 34 | 0.389 | AFSC |
| 184 | Groundfish | Commercial fishing permit holders | Cordova | east | Sablefish revenue | 1985 | 2018 | 34 | -0.260 | AFSC |
| 185 | Groundfish | Commercial fishing permit holders | Cordova | east | Sablefish pounds harvested | 1985 | 2018 | 34 | -0.182 | AFSC |
| 186 | Groundfish | Commercial fishing permit holders | Cordova | east | Pacific cod revenue | 1986 | 2018 | 33 | 0.276 | AFSC |
| 187 | Groundfish | Commercial fishing permit holders | Cordova | east | Pacific cod pounds harvested | 1986 | 2018 | 33 | 0.263 | AFSC |
|  |  |  |  |  |  |  |  |  |  |  |

Table S2. Model results from dynamic factor analysis of 187 biological time series spanning 2010-2018 and encompassing the 2014-2016 northeast Pacific marine heatwave. The top five ranking models are shown based on Akaike’s Information Criterion corrected for small sample sizes (ΔAICc). A total of nine models were run that included three different variance structures for the R matrix and three possible trends for each variance structure. Results shown are variance structure, number of trends tested, log likelihood (logLik) number of parameters (K), and ΔAICc.

| Variance structure | # of trends | logLik | K | ΔAICc |
| --- | --- | --- | --- | --- |
| same variance and covariance | 1 | -1867.4 | 193 | 0 |
| same variance, no covariance | 1 | -1871.6 | 192 | 5.8 |
| same variance and covariance | 2 | -1662.3 | 383 | 164.8 |
| same variance, no covariance | 2 | -1670.4 | 382 | 177.4 |
| different variance, no covariance | 1 | -1792.2 | 382 | 421.0 |


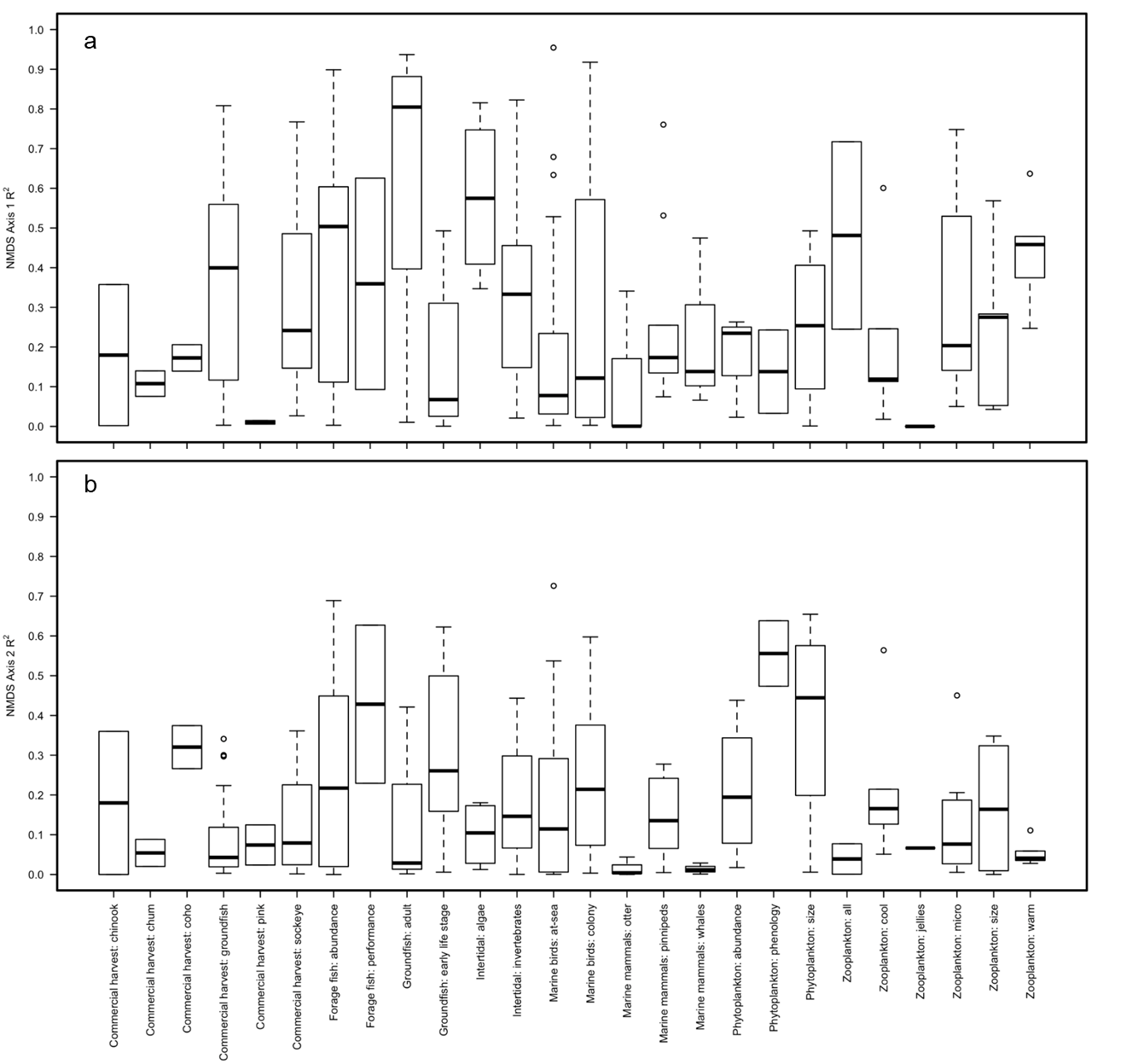


Figure S1. Correlation of biological time series by taxa and metric with (a) non-metric multidimensional scaling (nMDS) axis 1 and (b) nMDS axis 2 (bars depict median, boxes are first and third quartiles, whiskers extend to the most extreme data point up to 1.5 × the interquartile range beyond the box, and open circles are outliers).


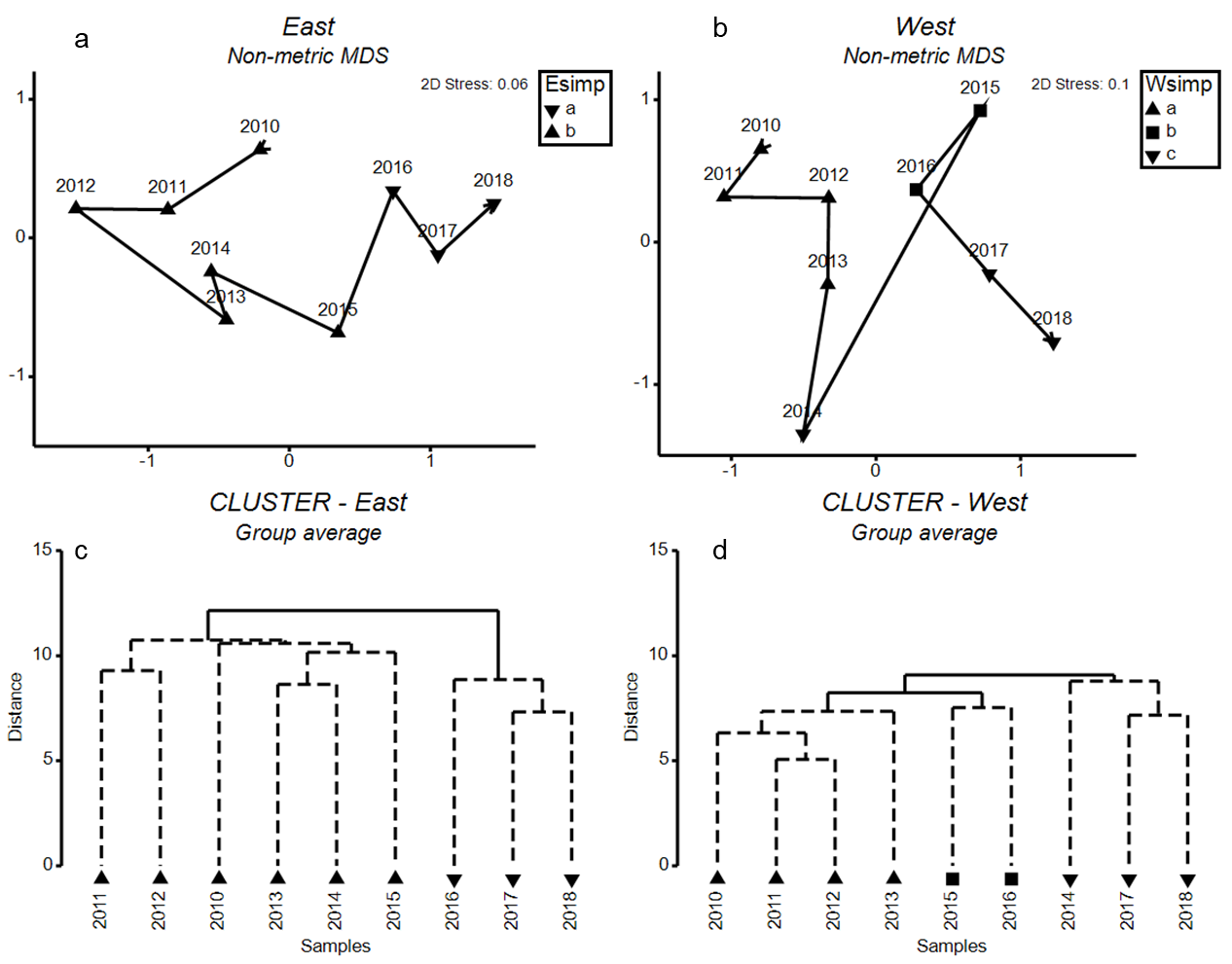


Figure S2. Comparison of the annual (a) east and (b) west Gulf of Alaska community from 2010-2018 by non-metric multi-dimensional scaling of normalized Euclidean distances; the vector connects each year through time. Cluster analyses of (c) east and (d) west show statistically significant groupings (solid lines in dendrogram); dashed lines connect grouped samples determined by SIMPROF and denoted by symbols and letter groups for east ‘Esimp’ and west ‘Wsimp’. For this analysis, we used a subset of time series (n = 93 out of 187) for available data specific to the East (n = 63) and West (n = 36) of the Gulf of Alaska (Table S1).

Figure S3. Cluster analyses of biological time series over a 19-year period prior to, during, and after the 2014-2016 marine heatwave in the Gulf of Alaska. In this analysis we used only time series (n = 87) that extended over a time period longer than was used in the analysis for Figure S2. These longer time series confirm that years following the 2014 onset of the PMH do indeed stand out from the others.

## Data Files

Three common separated text files (.csv) are provided. The file named “Suryan_et_al_GOA_synthesis_v5r3_alldata_ScientificReports_ESM.csv” contains all the time series that were included in the analyses for this paper, two header lines of notes, units of each metric, and DOIs for individual time series where available. The file “Suryan_et_al_GOA_synthesis_v5r3_orig_data_IDs_90_135to140_ScientificReports_ESM.csv” contains the original data of six time series where the linearly increasing trend was removed prior to analyses. “Suryan_et_al_GOA_synthesis_v5r3_Table_S1_ScientificReports_ESM.csv” contains all the information in Table S1 but in a format that can be sorted or filtered.”
